# Identification of integrated stress response-related prognostic genes in high-grade serous ovarian cancer using Mendelian randomization, single-cell RNA sequencing, and bulk RNA sequencing

**DOI:** 10.3389/fonc.2026.1798083

**Published:** 2026-05-05

**Authors:** Qian Li, Fanqiang Kong, Penghua Cui, Xiujuan Gao, Xinrong Zhuang, Zhongkai Zhang, Tian Tian, Guixiang Zhang

**Affiliations:** 1 Department of Gynecology, Affiliated Hospital of Chengde Medical University, Chengde, Hebei, China; 2 Department of Radiology, Affiliated Hospital of Chengde Medical University, Chengde, Hebei, China; 3 Department of Operating Room, Affiliated Hospital of Chengde Medical University, Chengde, Hebei, China

**Keywords:** high-grade serous ovarian cancer, integrated stress response, mendelian randomization, prognostic model, single-cell RNA sequencing

## Abstract

**Background:**

The integrated stress response (ISR) can cause high-grade serous ovarian cancer (HGSOC) cells to arrest in G1 phase, significantly suppressing their hyperproliferation. Nevertheless, the specific molecular mechanisms of the ISR in HGSOC remain unclear.

**Methods:**

This study integrated HGSOC- and ISR-related data. Prognostic genes were identified via differential expression, Mendelian randomization, and univariate Cox analyses. The risk model was constructed and evaluated using the risk score. Then, gene set enrichment analysis, drug sensitivity analyses, and single-cell RNA sequencing were used to explore risk model-related mechanisms, and reverse transcription quantitative polymerase chain reaction (RT-qPCR), western blotting, and immunohistochemical staining were applied to detect prognostic gene expression.

**Results:**

Four prognostic genes (*NUP35, CASP3, BAG5*, and *DNAJB1*) for HGSOC were identified. Using the risk score to classify patients into high- and low-risk groups, the model accurately predicted patients’ prognoses. The PPAR signaling pathway was significantly enriched in both risk groups. The half-maximum inhibition concentration of NPK76-II-72–1 was positively correlated with *DNAJB1 expression*. Moreover, *BAG5* and *DNAJB1* expression changed dynamically during epithelial cell differentiation. RT-qPCR, western blotting, and immunohistochemical staining demonstrated that *NUP35, CASP3*, and *BAG5* were upregulated in HGSOC, consistent with the results of Wilcoxon’s analysis.

**Conclusions:**

This study developed an ISR-associated prognostic model for HGSOC, which could improve patient prognosis.

## Background

Epithelial ovarian cancer is a malignant tumor that seriously threatens women’s health (1). High-grade serous ovarian cancer (HGSOC) is the most frequent histological subtype of epithelial ovarian cancer (approximately 75% of all cases) and the most aggressive and detrimental histological subtype (2). Current treatments for HGSOC include surgical resection, platinum-based chemotherapy, targeted therapy, and immunotherapy (3). Even with multimodal treatment, the overall 5-year survival rate is 31% because of drug resistance (4). Thus, exploring the pathogenesis of HGSOC at multiple levels and identifying relevant prognostic genes are essential for accurately predicting patient outcomes and developing personalized treatment strategies.

The integrated stress response (ISR) is a complex physiological response that occurs when the body encounters various stress factors (such as infection, hypoxia, and nutrient deficiency) through the coordinated and integrated response of multiple systems (5). The ISR acts as a “double-edged sword” in cancer, serving as both a survival tool for tumors to adapt to harsh microenvironments and a potential therapeutic target (6). Previous studies confirmed that the ISR promotes metastasis in hypoxic breast and cervical cancer cells, whereas it might exert tumor-suppressive effects in liver cancer by regulating lipid metabolism and oxidative stress (7, 8). Therefore, intervention strategies should be carefully selected according to the cancer type. However, little research has aimed to improve the outcomes of patients with HGSOC by targeting ISR-related genes. Therefore, further research on ISR-related prognostic genes during HGSOC development is essential for accurate prognostic prediction and specific targeted therapy in patients with HGSOC.

In recent years, Mendelian randomization (MR) and single-cell RNA sequencing (scRNA-seq) have provided novel perspectives for deciphering the etiological mechanisms of tumors. MR uses genetic variants as instrumental variables (IVs) to assess causal relationships between exposures and outcomes (9), and it has been increasingly applied in HGSOC research. Meanwhile, scRNA-seq reveals tumor heterogeneity, immune microenvironment regulation, and intercellular interactions in HGSOC at single-cell resolution (10). Based on these techniques, this study integrated transcriptomic data; focused on the ISR pathway; and employed MR, prognostic modeling, and single-cell analysis to explore the expression patterns, prognostic value, and potential regulatory roles of ISR-related genes (ISR-RGs) at the cellular level in HGSOC. The study aimed to provide a reference for subsequent functional studies and explorations of individualized treatment strategies.

## Methods

### Data acquisition

The transcriptome dataset, clinical data, and overall survival (OS) information of 365 HGSOC tumor samples in the TCGA-OV dataset were gathered from The Cancer Genome Atlas (TCGA, https://portal.gdc.cancer.gov/) (11). Next, the GSE102073 (GPL16791) dataset (12), which included 84 HGSOC tumor tissues with OS information, was fetched from the Gene Expression Omnibus (GEO) database (http://www.ncbi.nlm.nih.gov/geo/) (13). The GSE54388 (GPL570) dataset (14), containing 16 HGSOC tumor and 6 normal ovarian surface epithelium tissues, was retrieved from the GEO database (15). Then, the GSE154600 (GPL16791) scRNA-seq dataset, including five HGSOC tumor tissues, was obtained from the GEO database (13). Moreover, 534 ISR-RGs were acquired from the literature (16) (**Supplementary Table S1**). Specifically, these genes were screened and curated from the Gene Ontology (GO) database, and they represented a broad collection encompassing five core stress response subcategories: heat shock response, oxidative stress response, unfolded protein response, hypoxia stress response, and DNA damage response. Subsequently, genome-wide association summary statistics for candidate genes were collected from the IEU OpenGWAS database (https://gwas.mrcieu.ac.uk/). The HGSOC GWAS dataset (“ieu-a-1121”), which contained 13,037 case European samples, 40,941 control European samples, and 7,849,324 single-nucleotide polymorphisms (SNPs) was downloaded.

### Differential expression analysis

Using the limma package (V 3.56.2) (17), differentially expressed genes (DEGs) between HGSOC and normal ovarian tissues in the GSE54388 dataset were evaluated [P < 0.05, |log_2_ fold change (FC)| > 0.5]. The ggplot2 package (V 3.5.1) (18) was used to construct volcano plots. The ComplexHeatmap package (V 2.18.0) (19) was used to construct heatmaps.

### Identification, functional annotation, and protein-protein interactions (PPIs) of candidate genes

Using the ggvenn package (V 0.1.10) (20), the intersecting DEGs and ISR-RGs were identified as candidate genes. GO and Kyoto Encyclopedia of Genes and Genomes (KEGG) enrichment analyses were performed via the clusterProfiler package (V 4.10.1) using the criterion P.adjust < 0.05 (21). Protein associations in candidate genes were studied using the Search Tool for the Retrieval of Interacting Genes database (http://www.string-db.org/; (confidence = 0.4), and the PPI network was constructed using Cytoscape (V 3.7.2).

### MR analysis and biomarker identification

To investigate the causal relationship between candidate genes and HGSOC, the candidate genes were set as the exposures, HGSOC was set as the outcome, and SNPs were set as IVs. The two-sample MR analysis was based on three core assumptions: IVs were closely associated with the candidate genes; IVs were not associated with confounders; and IVs affected HGSOC solely through the candidate genes. The “extract instruments” function was used to select IVs using the following screening criteria: P < 5 × 10^−6^, clump = TRUE, r^2^ = 0.001, kb = 10, and variables with fewer than three SNPs or F-statistic < 10 were excluded.

The mr function from the TwoSampleMR package (V 0.6.4) (22) was applied, incorporating five algorithms, namely MR-Egger (23), weighted median (24), inverse variance weighted (IVW) (25), simple mode (26), and weighted mode (27), to perform the two-sample MR analysis. Candidate exposures were selected using the IVW algorithm (P < 0.05). The reliability of the MR results was assessed through heterogeneity tests, horizontal pleiotropy tests, and leave-one-out analyses. Heterogeneity was evaluated using the Q statistic and *I²* statistics, whereas the mr_presso function was employed to assist in detecting horizontal pleiotropy (P > 0.05 indicated no horizontal pleiotropy). MR Steiger analysis was further conducted to verify the direction of causality. Finally, the core genes identified through MR analysis were used in subsequent studies.

### Construction and confirmation of the risk model

Genes associated with HGSOC survival were identified from TCGA-OV dataset specimens using the survival package. Univariate Cox regression (survival package V 3.7-0) (28) (hazard ratio [HR] ≠ 1, P < 0.1) (29) and the proportional hazards (PH) hypothesis test (cox.zph function, P > 0.05) were applied. Prognostic genes were selected on the basis of consistent odds ratios (ORs) in MR and consistent HRs in Cox regression.

The expression of prognostic genes in the HGSOC and normal ovarian tissue samples were compared using the Wilcoxon test (P < 0.05). Thereafter, in the TCGA-OV and GSE102073 datasets, the randomForestSRc package (V 3.3.1) (30) was used to calculate each patient’s risk score. A random survival forest (RSF) model was built using the identified prognostic genes (ntree = 20, mtry = 1). The survminer package computed the optimal cutoff for the risk score, which was used to construct the risk model. The ggplot2 package (V 3.5.1) was used to present the risk score and survival status distributions. Thereafter, HGSOC samples in the TCGA-OV and GSE102073 datasets were divided into high- and low-risk groups based on the calculated risk score cutoff. The survminer package was used to generate Kaplan–Meier curves to assess survival differences (P < 0.05). The survivalROC package (31) was employed to create ROC curves to verify the risk model (area under the curve [AUC] ≥ 0.6). To evaluate the relationship between risk scores and prognostic genes, the surv_cutpoint() function was used to divide the samples from the TCGA-OV and GSE102073 datasets into high- and low-expression groups based on the median expression of each prognostic gene. The Wilcoxon test was applied to compare the distribution differences in risk scores between the high- and low-expression groups (P < 0.05). Finally, the ggplot2 (V 3.5.1) package was used for visual display.

### Prognostic factors and clinical features analyses

To identify factors associated with prognosis using the TCGA-OV dataset, univariate Cox analysis (HR ≠ 1, P < 0.05) was conducted using the survival package in consideration of the risk score and clinical features. Then, a PH assumption test (P > 0.05) was performed using the cox.zph function. To explore the link between clinical features and the risk score, the Wilcoxon or Kruskal–Wallis test was used to evaluate score differences among clinical feature subgroups in TCGA-OV (P < 0.05).

### Gene set enrichment analysis (GSEA) and immune microenvironment analysis

To uncover KEGG pathways distinguishing the high- and low-risk groups via GSEA, the relevant gene set (c2.cp.kegg.v7.5.1.symbols.gmt) was downloaded from the Molecular Signatures Database. Differential expression analysis (high-risk group vs. low-risk group in TCGA-OV) was performed using DESeq2 (V 1.42.0) (32). Then, clusterProfiler (V 4.10.1) (21) was used to conduct GSEA using the following enrichment criteria: |normalized enrichment score| > 1, P.adjust < 0.05, and false discovery rate (FDR) < 0.25.

To assess immune infiltration in TCGA-OV, the CIBERSORT algorithm in the immunedeconv package (V 2.1.0) (33) was used to compute the frequencies of 22 immune cell types in HGSOC specimens. Then, the differences in the immune cell distribution between the high- and low-risk groups were compared using the Wilcoxon test, and the Benjamini–Hochberg method was applied to correct for multiple comparisons to control the FDR (P.adjust < 0.05).

### Immunotherapy and tumor mutation analysis

To explore immune checkpoint inhibitor (ICI) expression in the high- and low-risk groups of the TCGA-OV dataset, the Wilcoxon test was used to evaluate expression differences for 20 ICIs (34) (P < 0.05). For patients with HGSOC, the Tumor Immune Dysfunction and Exclusion (TIDE) platform (tide.dfci.harvard.edu/login/) was used to compute scores, and the Wilcoxon test was used to compare TIDE scores between the risk groups (P < 0.05).

To understand somatic mutations between the high- and low-risk groups, the tumor mutational burden data of patients with HGSOC were downloaded from TCGA-OV. The maftools package (V 2.18.0) (35) was used to create a waterfall plot of the top 20 mutated genes.

### Drug sensitivity analysis

To assess the chemotherapeutic drug sensitivity of patients with HGSOC, 138 drugs were obtained from Genomics of Drug Sensitivity in Cancer (https://www.cancerrxgene.org/). The pRRophetic package (V 0.5) (36) was employed to calculate the half-maximum inhibition concentration (IC_50_) values of the drugs. The psych package (V 2.4.3) was used to perform Spearman’s analysis to probe the link between the prognostic genes and the IC_50_ of each drug (|cor| > 0.3, P < 0.05).

### Regulatory network and analysis of prognostic gene expression at the protein level

To explore transcription factors (TFs) regulating prognostic genes, the NetworkAnalyst database (https://www.networkanalyst.ca) was used to predict relevant TFs, and Cytoscape (V 3.7.2) was used to create the regulatory network. To gain an understanding of prognostic gene expression at the protein level, the GEPIA database (gepia.cancer-pku.cn/) was employed to study their expression in different types of cancers.

### scRNA-seq analysis

To obtain high-quality cells, the Seurat package (V 4.1.0) (37) was used to analyze the single-cell data of GSE154600. Cells were selected on the basis of prespecified criteria (percent.mt < 10%, 399 < nFeature RNA < 4132, nCount RNA < 18,667) and then filtered (cells with fewer than 200 genes, genes covered by fewer than three cells, cells with less than 5% of all genes, cells with more than 95% of all genes, and cells with mRNA counts exceeding those in 95% of all cells). Data were standardized (LogNormalize function) and integrated to remove batch effects (harmony function). Then, 2000 highly variable genes (HVGs) were selected (FindVariableFeatures function), and principal components (PCs) were determined (RunPCA function, P < 0.05). Clustering (resolution = 0.1) was performed (FindClusters function), and the results were presented via Uniform Manifold approximation and Projection.

To distinguish cell types, marker genes from the literature (38) and FindAllMarkers (logfc.threshold = 0.25, min.pct = 0.1, only.pos = TRUE) were used with the CellMarker database (http://bio-bigdata.hrbmu.edu.cn/CellMarker/) for annotation, and a bubble plot was generated.

### Identification and functional analysis of key cells

To study prognostic gene expression in different cell types, the DotPlot function was used. Key cells were identified as those expressing multiple prognostic genes. To shed light on the function of different cell types, the ReactomeGSA package (V 1.16.1) (39) was exploited to analyze the functional enrichment of distinct cell subtypes.

### Cellular communication and pseudotime trajectory analyses

In an effort to infer the interactions between various cell types, the CellChat package (V 1.6.1) (40) was employed to conduct paired comparisons between different cell types in the HGSOC samples from the GSE154600 dataset. To explore key cell developmental pathways, the Monocle package (V 2.30.1) (41) analyzed pseudotime trajectories. Then, the plot_genes_in_pseudotime function studied prognostic genes’ expression trends.

### Reverse transcription quantitative polymerase chain reaction (RT-qPCR), western blotting, and immunohistochemical staining

Tumor tissues and adjacent normal tissues were collected from five patients with HGSOC at the Affiliated Hospital of Chengde Medical University. This research project was approved by the Ethics Committee of the Affiliated Hospital of Chengde Medical University (CYFYLL2025430), and patients provided written informed consent.

Total RNA from tissue samples was isolated using TRIzol (Invitrogen) following the manufacturer’s protocol. Total RNA was reverse-transcribed into cDNA using the SweScript First Strand cDNA synthesis kit (Servicebio) following the manufacturer’s directions. RT-qPCR was conducted using 2× Universal Blue SYBR Green qPCR Master Mix (Servicebio). The primer sequences for qPCR are presented in **Supplementary Table S2**. GAPDH served as an internal reference gene. The 2^−ΔΔCt^ method was employed to calculate the expression of prognostic genes. Additionally, GraphPad Prism 10 was utilized to illustrate the differences in the expression of prognostic genes between HGSOC and adjacent normal tissue samples (P < 0.05).

Proteins were extracted using RIPA lysis buffer (Servicebio) containing protease and phosphatase inhibitors (Servicebio). After quantification by the BCA assay (Servicebio), equal amounts of protein (20–40 µg) were denatured, separated by 10% SDS-PAGE (Servicebio), and transferred onto PVDF membranes (MilliporeSigma). The membranes were blocked with 5% (w/v) skim milk powder in Tris-buffered saline containing 0.1% Tween-20 (TBST, pH 7.4) for 1 h at room temperature. Subsequently, the membranes were incubated overnight at 4°C with the following primary antibodies diluted in blocking buffer: anti-NUP35 (1:1000, ABclonal), anti-CASP3 (1:1000, HUABIO), anti-DNAJB1 (1:1000, ABclonal), and anti-BAG5 (1:1000, HUABIO). An anti-GAPDH antibody (1:5000, Servicebio) was used in parallel as the loading control. All dilutions were prepared according to the manufacturers’ instructions or optimized protocols. After three washes with TBST, the membranes were incubated with a horseradish peroxidase (HRP)-conjugated secondary antibody (1:15,000, Servicebio) for 1 h at room temperature. Following additional washes, protein bands were visualized using an ECL substrate on a chemiluminescence imaging system (Bio-Rad) and quantified using ImageJ software.

The protein expression of the prognostic genes was assessed by immunohistochemistry as previously described (42). Briefly, ovarian tissue samples were fixed in 4% paraformaldehyde (Servicebio), embedded in paraffin, and sectioned at a thickness of 5 μm. After dewaxing and rehydration, antigen retrieval was performed via microwave heating. Sections were then blocked with 10% goat serum for 1 h and then incubated overnight at 4 °C with the following primary antibodies: anti-NUP35 (1:100, Zen-bioscience), anti-BAG5 (1:100, ABclonal), anti-DNAJB1 (1:100, HUABIO), and anti-CASP3 (1:500, HUABIO). After washing, the sections were incubated with corresponding HRP-conjugated secondary antibodies (ZSGB-Bio) at 37 °C for 1 h, followed by color development with DAB chromogen (Servicebio). Nuclei were counterstained with hematoxylin, and images were captured using an optical microscope (Zeiss).

### Statistical analysis

Data analysis and processing were fulfilled using R software version 4.2.2. Group comparisons were performed using the Wilcoxon and Kruskal–Wallis tests. Statistical significance was indicated by P < 0.05.

## Results

### Identification, functional enrichment, and PPI network of candidate genes in HGSOC

**Figure 1:**
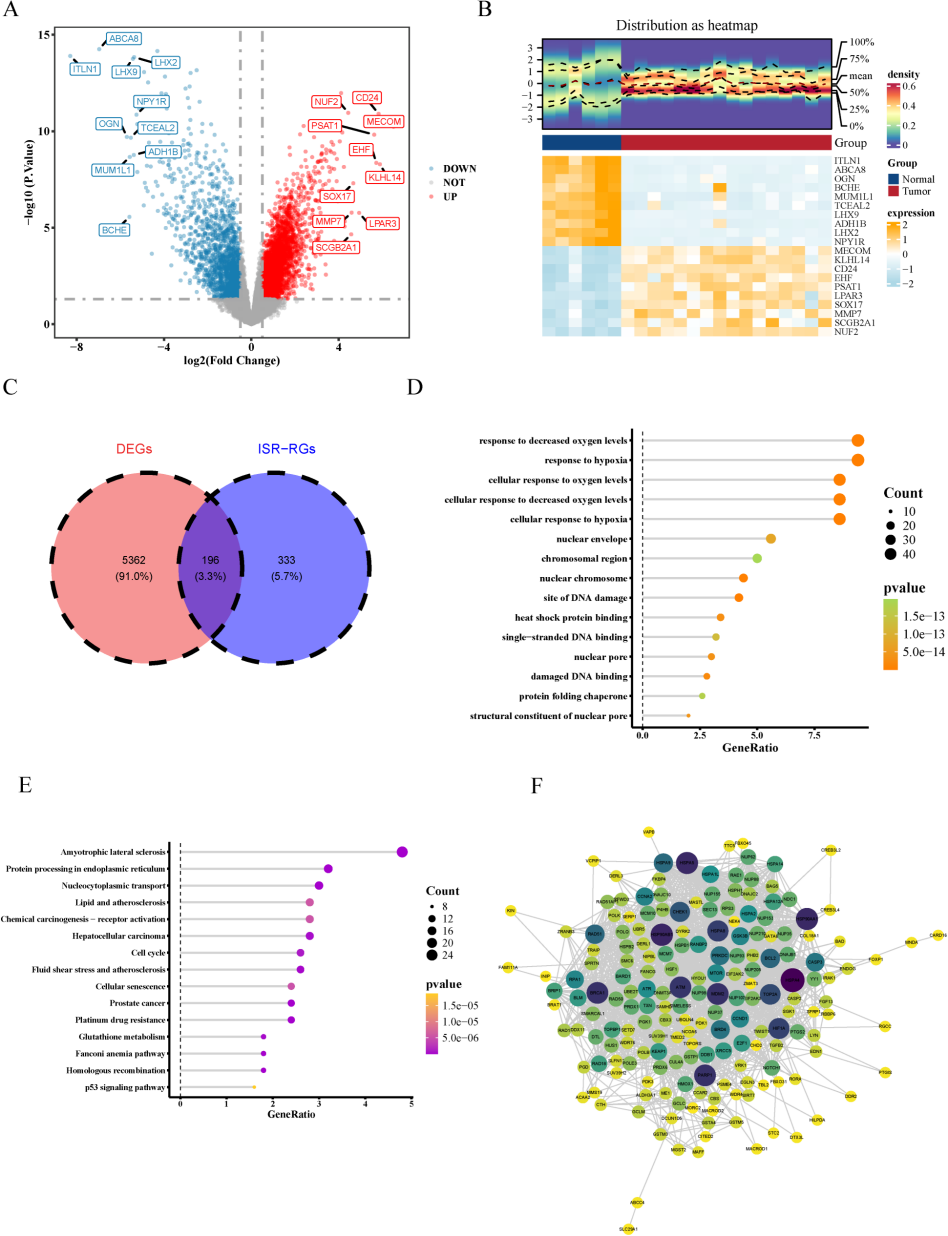
Identification, functional enrichment, and PPI network of candidate genes in HGSOC. **(A)** Volcano plot of differential expression analysis. Red and blue dots represent the top 10 upregulated and downregulated genes (sorted by log_2_ FC value), respectively. **(B)** Heatmap of differential expression. Upper section: Expression density heatmap of the top 10 upregulated and downregulated genes in the tumor group. Lower section: Clustered expression heatmap of these differentially expressed genes (DEGs). **(C)** Venn diagram of candidate genes. A total of 196 overlapping genes were identified as candidate genes through cross-dataset comparisons. **(D)** GO enrichment analysis of candidate genes. **(E)** KEGG pathway enrichment analysis. Top 15 enriched signaling pathways were ranked based on statistical significance. **(F)** PPI network of 185 candidate gene.

In the GSE54388 dataset, 5558 DEGs were identified between HGSOC and normal ovarian tissue specimens. Specifically, in HGSOC specimens, 3840 DEGs were upregulated, whereas 1718 DEGs were downregulated. The top 10 upregulated and downregulated DEGs, ranked according to log_2_ FC, were marked on a volcano plot. Meanwhile, the expression of these genes are presented in a heatmap in **Figures 1A, B**. Overall, 196 candidate genes were pinpointed by intersecting the 5558 DEGs with the 534 ISR-RGs (**Figure 1C**).

The 196 candidate genes were involved in 1174 GO terms (1030 biological processes, 55 cellular components, and 89 molecular functions and 72 KEGG pathways; **Figures 1D, E**; **Supplementary Tables S3**, **S4**). GO terms and KEGG pathways included damage checkpoint signaling and platinum drug resistance. The PPI network highlighted interactions among 185 genes, such as *CARD16*–*MNDA* and *SLC29A1*–*ABCC4* (**Figure 1F**).

### Identification of core genes in HGSOC

The 73 candidate genes significantly causally associated with HGSOC were identified by MR analysis dominated by the results of the IVW algorithm (P < 0.05). Among the 73 candidate genes, 33 genes (e.g., *BRD4*, *RPS3*) were risk factors for HGSOC occurrence (OR > 1), whereas 40 genes (e.g., *SAMHD1*, *RANBP2*) were protective factors for the development of HGSOC (OR < 1, **Supplementary Figure S1**). The scatter diagrams and forest plots of the SNP–exposure and SNP–HGSOC effects demonstrated a high extent of conformity between the study results and IVW results (**Supplementary Figures S2**, **S3**). Furthermore, the funnel plot highlighted the roughly symmetrical distribution of SNPs among the 73 candidate genes, indicating no significant bias (**Supplementary Figure S4**). The heterogeneity test result indicated a lack of heterogeneity for 40 candidate genes (P > 0.05, **Supplementary Table S5**). The results of the horizontal pleiotropic test similarly indicated that confounding factors did not exist in this investigation for 40 candidate genes (P > 0.05, **Supplementary Table S6**). By successively individual SNPs, the impact of the remaining SNPs on the HGSOC variables did not substantially change, indicating that the findings of the MR analysis of the 40 candidate genes were credible and unwavering (**Supplementary Figure S5**). The Steiger test findings demonstrated that the direction results of the 40 candidate genes were true and significant at P < 0.05, suggesting that the 40 candidate genes have a causal relationship with HGSOC (**Supplementary Table S7**). The 40 candidate genes were selected as core genes for subsequent analyses.

### Development and verification of risk models

**Figure 2:**
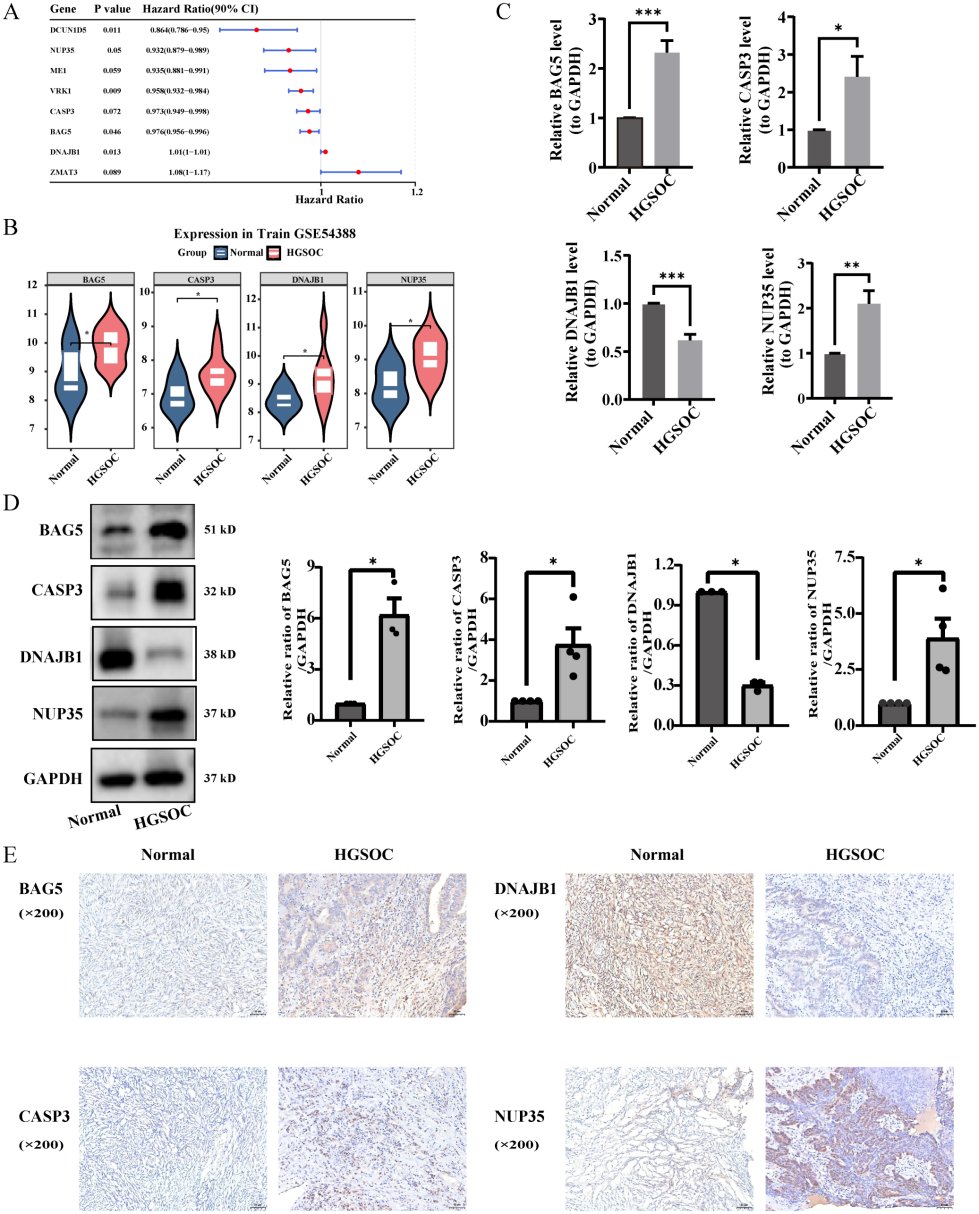
Development and verification of risk models. **(A)** A total of 8 survival- related genes were obtained through univariate Cox analysis (HR ≠ 1, *P* < 0.1). **(B)** The Wilcoxon test showed that the four prognostic genes were significantly upregulated in HGSOC samples. **(C–E)** Experimental validation of *NUP35*, *CASP3*, *BAG5, and DNAJB1* expression in HGSOC and normal ovarian tissue samples using RT-qPCR **(C)**, western blotting **(D)**, and immunohistochemical staining **(E)**. Data are presented as mean ± SD; *P < 0.05, **P < 0.01, ***P < 0.001..

After performing the PHs assumption test on 39 genes (P > 0.05, **Supplementary Table S8**), eight genes associated with the prognosis of HGSOC were identified through univariate Cox analysis (HR ≠ 1, P < 0.1, **Figure 2A**). By further integrating MR analysis, four prognostic genes (*NUP35, CASP3, BAG5,* and *DNAJB1*) were identified, among which *NUP35, CASP3*, and *BAG5* were protective factors, and *DNAJB1* was a risk factor. The Wilcoxon test illustrated that all four genes were significantly upregulated in HGSOC samples from the GSE54388 dataset (P < 0.001, **Figure 2B**). The RT-qPCR, western blotting, and immunohistochemical staining results demonstrated that *NUP35, CASP3*, and *BAG5* were significantly upregulated in HGSOC samples, consistent with the Wilcoxon test results. Conversely, *DNAJB1* expression exhibited a downward trend in both validation methods, contradicting the results of the database analysis (**Figures 2C–E**).

**Figure 3:**
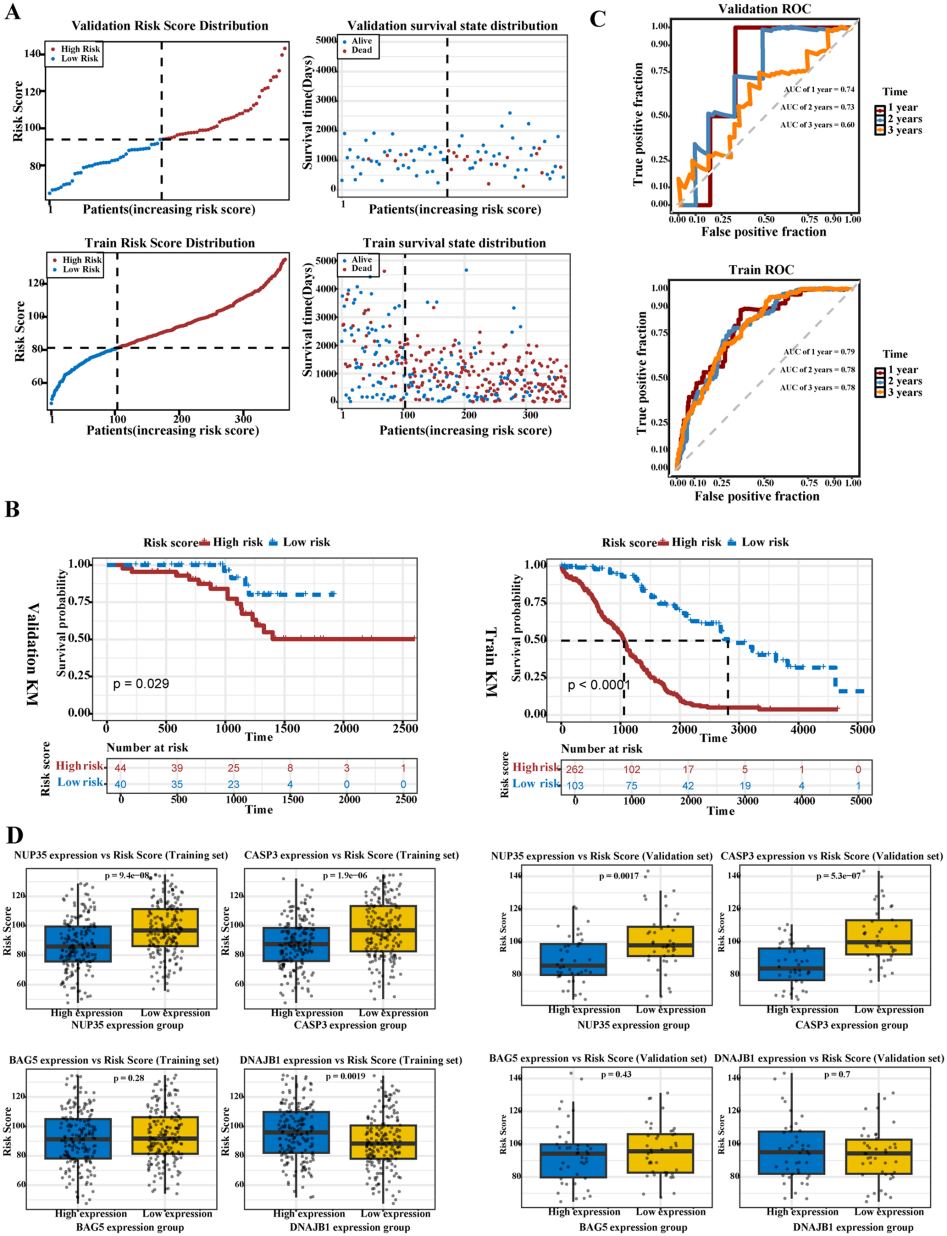
Prognostic performance and validation of the risk model. **(A)** The risk score distribution plots (top-left, bottom-left) and survival status distribution plots (top-right, bottom-right) for the validation and training sets demonstrate the effectiveness of the prognostic model, which can predict patients’ survival time and status based on risk scores (Each dot represents a sample: Red indicates high risk or death, while blue represents low risk or survival). **(B)** The Kaplan–Meier curve analysis of validation set (left) and training set (right) showed that the survival probability of the high-risk group is significantly lower than that of the low-risk group (P < 0.05). **(C)** The ROC curve analysis method demonstrates the predictive performance of the validation set (left) and the training set (right) for patients’ survival status at different time points (1, 2, and 3 years). **(D)** In the TCGA-OV dataset and the GSE102073 dataset, the risk score differences of prognostic genes (*NUP35, CASP3, DNAJB1*, and *BAG5*) between high and low expression groups..

Based on the optimal cutoff of the risk score calculated by the RSF model, samples in the TCGA-OV (81.16695) and GSE102073 (94.03832) datasets were divided into high- and low-risk groups. The risk curve and survival status distribution indicated that the mortality rate was lower in the low-risk group, whereas survival outcomes were worse in the high-risk group (**Figure 3A**). Kaplan–Meier survival curves confirmed that the survival rate was significantly lower in the high-risk group than in the low-risk group in both the TCGA-OV (P < 0.0001) and GSE102073 (P = 0.029) datasets (**Figure 3B**). ROC curves indicated that the model exhibited moderate predictive performance for 1-, 2-, and 3-year OS (AUC ≥ 0.6, **Figure 3C**).

The relationship between the risk score and the expression of prognostic genes was further analyzed using the Wilcoxon test. In the TCGA-OV dataset, the risk score was higher in the high-expression groups of *NUP35* and *CASP3*, whereas it was significantly lower in the high-expression group of *DNAJB1* (P < 0.05). In the GSE102073 dataset, the risk score was also higher in the high-expression groups of *NUP35* and *CASP3* (P < 0.05, **Figure 3D**). These results indicated that the associations of *NUP35, CASP3*, and *DNAJB1* with the risk score were consistent across cohorts, suggesting their potential value as prognostic stratification biomarkers.

### Independent prognostic factors, clinical characteristics, pathway enrichment, and immune infiltration analyses in HGSOC

**Figure 4:**
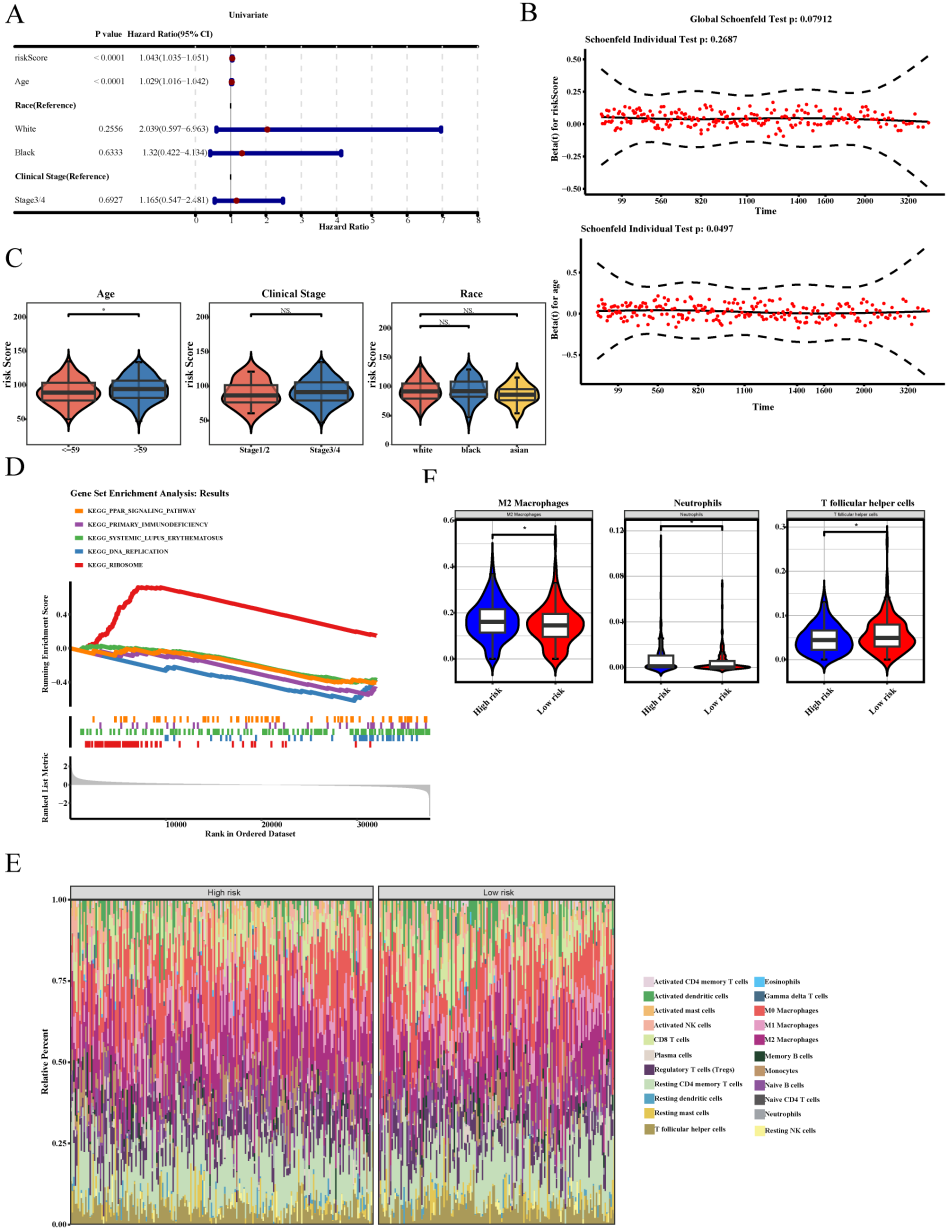
Independent prognostic factors, clinical characteristics, pathway enrichment, and immune infiltration analyses in HGSOC. **(A)** The relative risk (HR) values of risk score and clinical features by univariate Cox regression analysis (HR ≠ 1 and P < 0.05). **(B)** The PH assumption test for risk score and age (P > 0.05). **(C)** Differential analysis in risk scores among subgroups with distinct clinicopathological characteristics (P < 0.05). **(D)** GSEA Enrichment Analysis Plot. The top five broken lines are the broken lines of gene EnrichmentScore. The middle barcode-like parts are Hits, each vertical line corresponding to a gene under the gene set. The bottom is the rank value distribution chart of all genes. **(E)** 22 kinds of immune infiltration cells abundance heat map. The upper color bar categorizes samples into high-risk group (red) and low-risk group (blue). On the right side of the heatmap are the cell types, and the color gradient in the heatmap represents cell proportions, with yellow indicating high proportions and blue indicating low proportions. **(F)** Box plots of immune cell differences showed significant differences between high-risk group and low-risk group in M2 Macrophages, Neutrophils, and T follicular helper cells (P < 0.05). * P<0.05.

Univariate Cox regression analysis illustrated that age and the risk score were significantly associated with the prognosis of HGSOC (HR ≠ 1, P < 0.05, **Figure 4A**), and the PHs assumption test further confirmed that the risk score was associated with patient prognosis (P > 0.05, **Figure 4B**). In addition, the risk score significantly differed among the age subgroups in the TCGA-OV dataset (P < 0.05). These results indicated a potential correlation between the risk score and patient age (**Figure 4C**).

GSEA revealed that 15 pathways were differentially enriched between the high- and low-risk groups, including the PPAR signaling pathway and beta-alanine metabolism (**Figure 4D**; **Supplementary Table S9**). Immune infiltration analysis demonstrated that M2 macrophages, neutrophils, and T follicular helper cells exhibited significant differences in infiltration between the two groups (P < 0.05). Among them, the infiltration levels of neutrophils and M2 macrophages were lower in the low-risk group, whereas that of T follicular helper cells was lower in the high-risk group (**Figures 4E, F**).

### Immunotherapy, tumor mutations and drug sensitivity, regulatory networks, and protein-level expression of the prognostic genes in HGSOC

**Figure 5:**
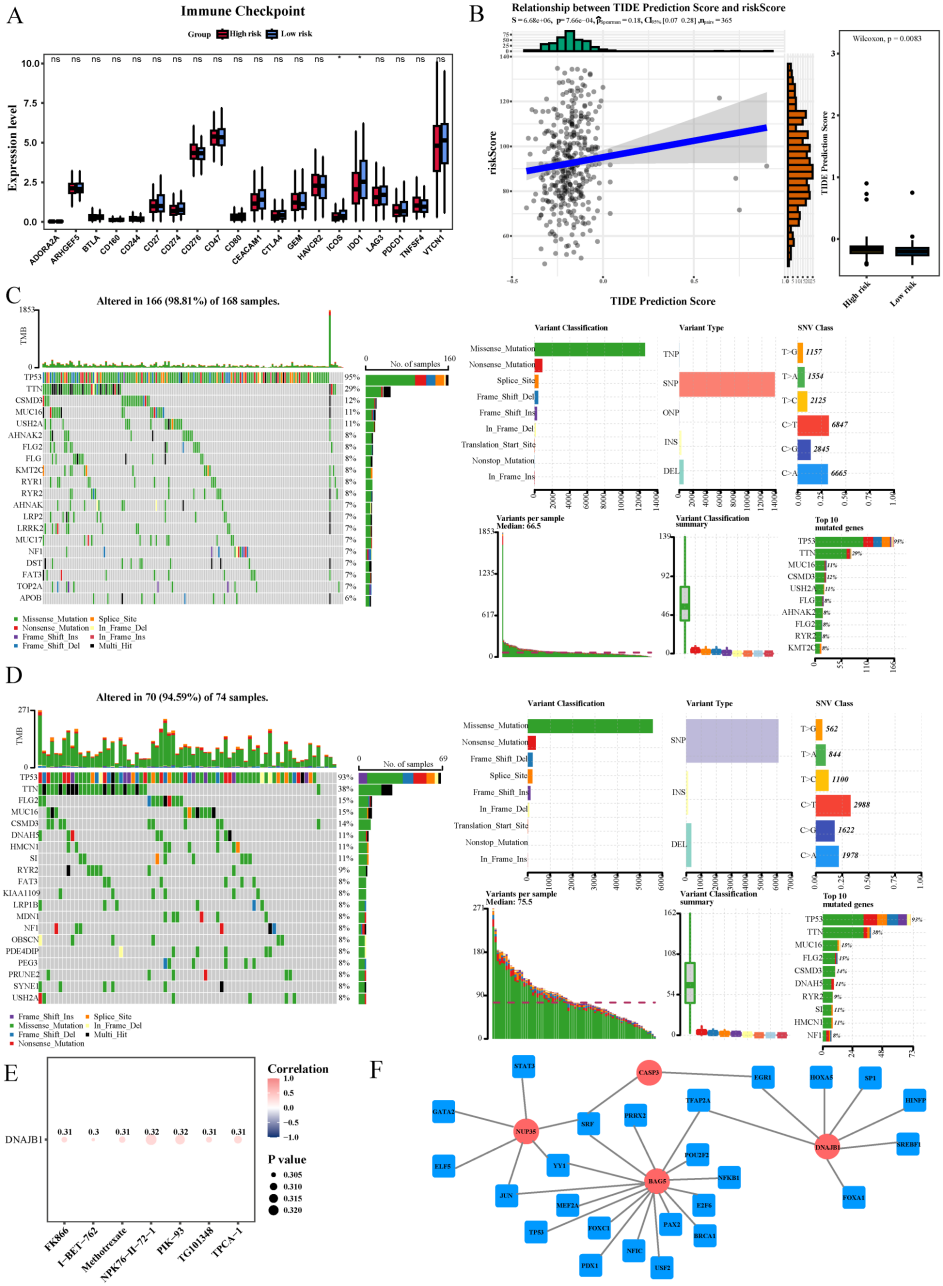
Immunotherapy, tumor mutations and drug sensitivity, regulatory networks, and protein-level expression of the prognostic genes in HGSOC. **(A)** Boxplot of immune checkpoint expression showing 2 immune checkpoint genes (*ICOS* and *IDO1*) significantly different between the high- and low-risk groups (P < 0.05). **(B)** TIDE analysis showed significant differences between the high- and low-risk groups (right), and risk scores were positively correlated with TIDE predictive scores (left), indicating that high-risk samples responded more strongly to immune checkpoint blockade. **(C, D)** Mutation distribution waterfall plots showed the TOP3 gene with the highest mutation frequency in high-risk group **(C)** and low-risk group **(D)**. **(E)** Drug sensitivity analysis of four prognostic genes. F TF-mRNA regulatory network consists of 25 transcription factors and 4 prognostic genes, among which *BAG5* predicts the most transcription factors. Transcription factor SRF interacts with prognostic genes *BAG5*, *NUP35* and *CASP3*..

Significant differences were observed in the expression of two immune checkpoint molecules (*ICOS* and *IDO1*) between the high- and low-risk groups (P < 0.05, **Figure 5A**). Furthermore, the TIDE score also significantly differed between the two groups (P = 0.0083, **Figure 5B**), suggesting the potential value of immunotherapy in HGSOC.

Mutation analysis revealed that the genes with the highest mutation frequencies in the high-risk group were *TP53* (95%), *TTN* (29%), and *CSMD3* (12%), whereas those in the low-risk group were *TP53* (93%), *TTN* (38%), and *FLG2* (15%). The most common mutation type of missense mutation (**Figures 5C, D**).

Drug sensitivity analysis demonstrated that the IC_50_s of NPK76-II-72–1 and PIK-93 were positively correlated with *DNAJB1* expression (cor = 0.32, P < 0.05, **Figure 5E**; **Supplementary Table S10**), suggesting that these two drugs exert therapeutic effects through *DNAJB1*.

In total, 25 TFs were predicted using the four biomarkers. Among them, *BAG5*, *NUP35*, and *CASP3* were simultaneously regulated by SRF, and *DNAJB1* and *CASP3* were simultaneously regulated by *EGR1* (**Figure 5F**; **Supplementary Table S11**). At the protein level, *DNAJB1* was highly expressed in multiple cancers (**Supplementary Figure S6**). These results suggested that *SRF* and *EGR1* could affect the function of prognostic genes and participate in the pathogenesis of HGSOC.

### Identification and function of key cells in HGSOC

**Figure 6:**
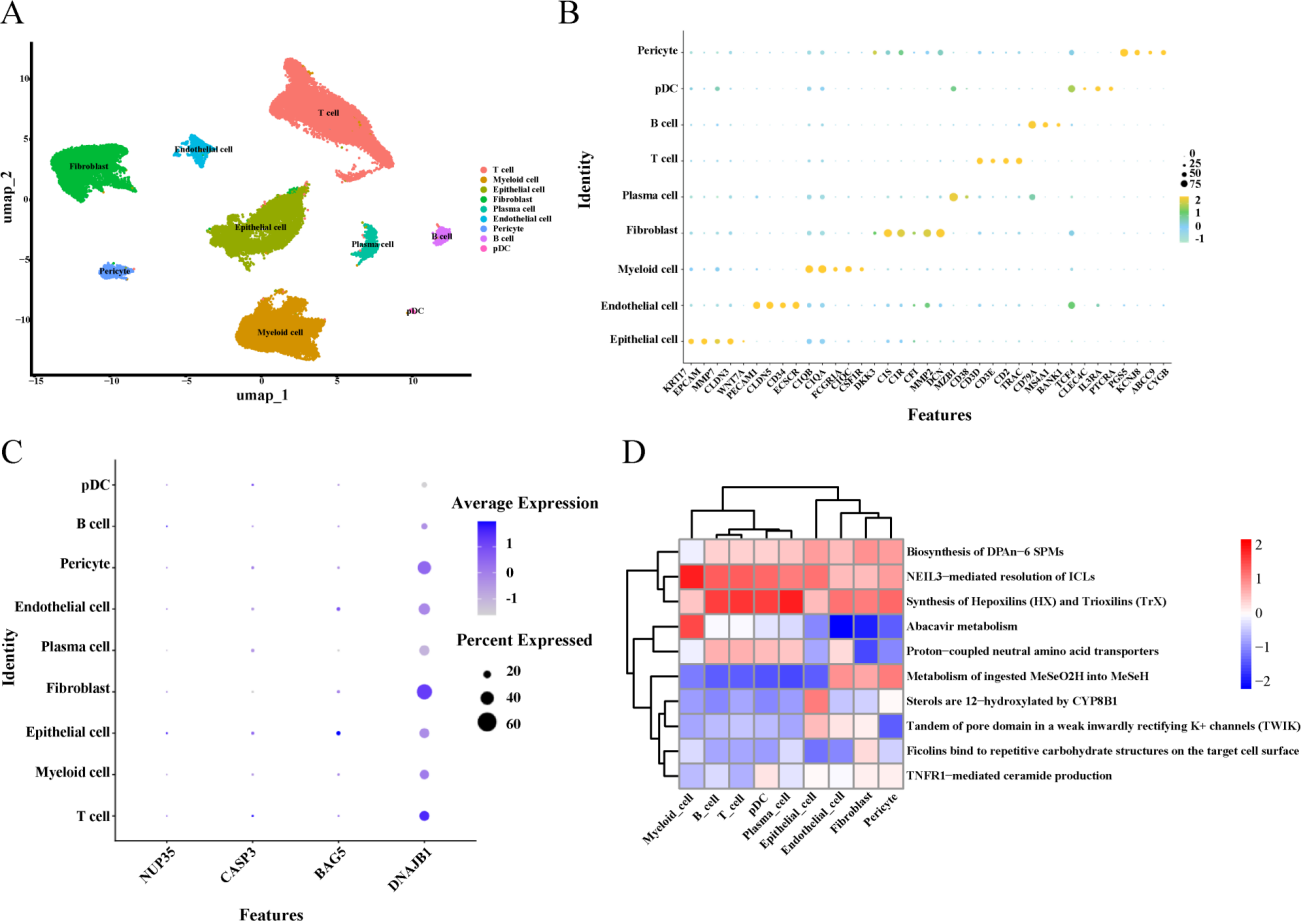
Cell annotation of single cell subclusters, identification of key cells and enrichment analysis. **(A)** UMAP map of 10 cell clusters annotated into 9 different cell types. **(B)** Bubble chart indicated the expression of marker genes representing various cell types in various clusters. **(C)** Bubble chart of expression of 4 prognostic genes in various cells. Darker colors indicate higher gene expression values, while larger dots represent greater gene expression prevalence. **(D)** Functional enrichment analysis of cells. The color gradient from blue to red indicates an increase in activity scores, with higher scores representing stronger pathway activation.

The scRNA-seq dataset included 51,643 cells and 23,885 genes (**Supplementary Figure S7**). After filtering, 40,565 cells and 23,885 genes were retained in the dataset (**Supplementary Figure S8**). The data were standardized to extract 2000 HVGs (**Supplementary Figure S9**), and 10 cell clusters were obtained via clustering analysis of the top 30 PCs (**Supplementary Figures S10**–**S12**). The 10 cell clusters were annotated into nine different cell types: T cells, B cells, myeloid cells, epithelial cells, endothelial cells, fibroblasts, pericytes, plasma cells, and plasmacytoid dendritic cells (**Figure 6A**). The bubble chart in **Figure 6B** presents the expression of marker genes in the nine cell types. The four prognostic genes (*BAG5*, *NUP35*, *DNAJB1,* and *CASP3*) exhibited elevated expression in epithelial cells (**Figure 6C**). Thus, epithelial cells were selected as key cells for subsequent analysis. The functional analysis indicated that NEIL3-mediated resolution of interstrand DNA–DNA crosslinks and 12-hydroxylation of sterols by CYP8B1 were significantly enriched in epithelial cells in the HGSOC samples (**Figure 6D**).

### Cellular communication and pseudotime trajectory in HGSOC

**Figure 7:**
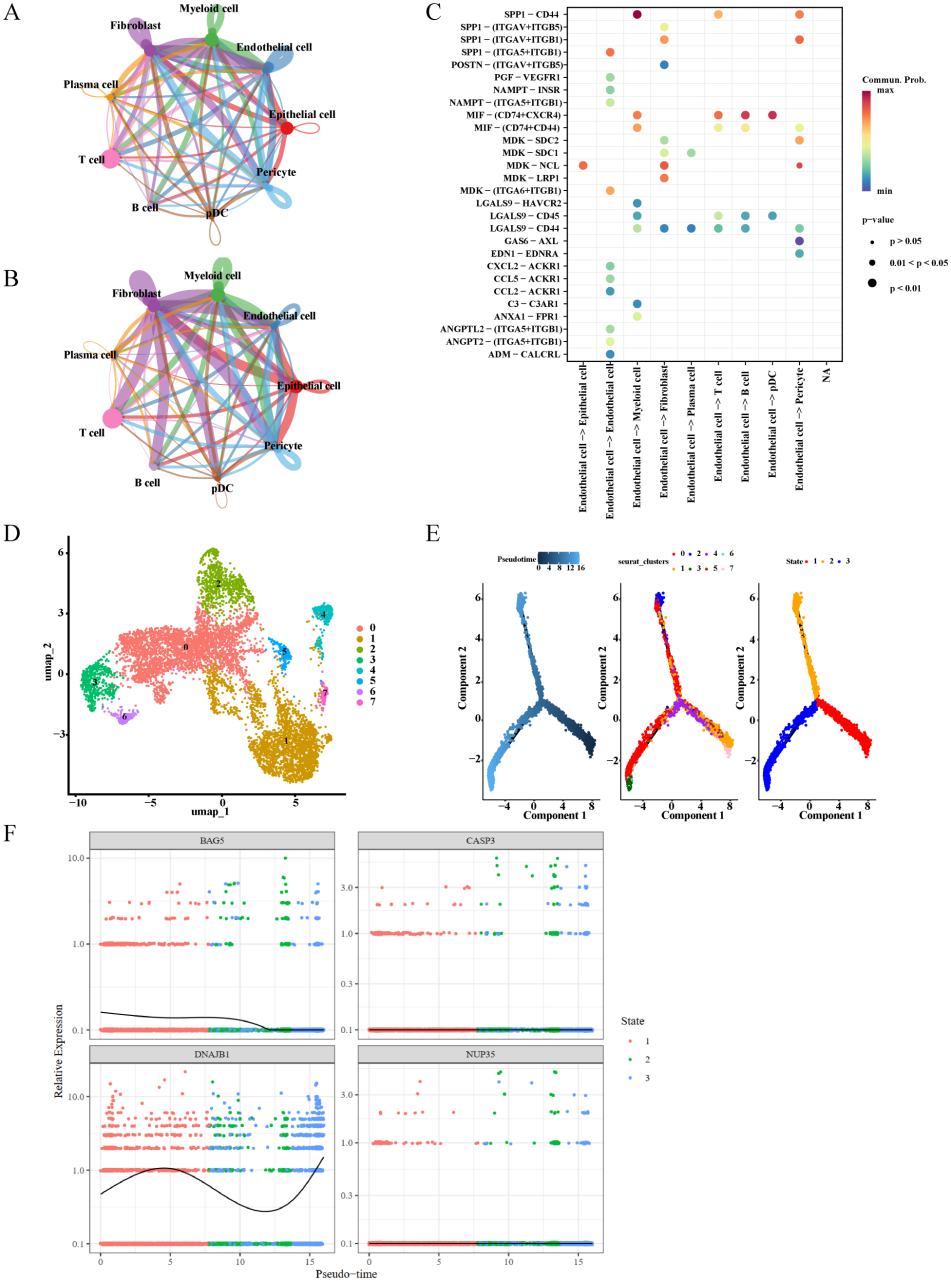
Cellular communication and pseudotime trajectory in HGSOC. **(A, B)** Cell-cell communication network of 9 cell types (depicted based on interaction count in figure **(A)** and interaction weight in figure **(B)**, where thicker lines indicate stronger cellular interacions. **(C)** The bubble diagram of ligand-receptor interaction between different cells. The abscissa is the communication relationship between different types of cells, and the ordinate is the corresponding ligand-receptor when two cells mediate. **(D, E)** The UMAP diagram of Epithelial Cell subgroup clustering. Epithelial Cells were divided into 8 subgroups [**(D)**, with different colors representing distinct cell types] and 3 differentiation states [**(E)**, each state marked by a different color]. **(F)** Expression dynamics plot of prognostic genes in pseudotime analysis.

Cell communication analysis revealed that interactions of epithelial cells with fibroblasts, pericytes, myeloid cells, and endothelial cells occurred more frequently and exhibited greater intensity in HGSOC samples (**Figures 7A, B**). Molecular-level analysis indicated that the main signal transmitted from endothelial cells to epithelial cells was the MDK–NCL pathway (**Figure 7C**), suggesting that epithelial cells participate in the pathogenesis of HGSOC through interactions with other cell types.

Pseudotime trajectory analysis indicated that epithelial cells differentiated from right to left over time into three distinct states (**Figures 7D, E**). During this process, *BAG5* expression initially decreased and then remained stable, whereas *DNAJB1* expression first increased, then decreased, and subsequently increased again. By contrast, *CASP3* and *NUP35* expression remained largely unchanged (**Figure 7F**), suggesting that these four prognostic genes might be associated with the process of epithelial cell differentiation.

## Discussion

In this study, we screened four key prognostic genes, namely *NUP35*, *CASP3*, *BAG5*, and *DNAJB1*, through MR, univariate Cox analysis, and PH hypothesis testing. We constructed an independent prognostic risk model for patients with HGSOC and calculated the risk scores. The model demonstrated similar performance in the GEO and TCGA datasets, indicating a certain degree of external data consistency.

In this study, we focused on *BAG5* and *DNAJB1*, both of which belong to the chaperone protein family and represent key components of the ISR regulatory network (43). Previous studies indicated that *BAG5* is overexpressed in certain tumor cells, in which it assists tumor cells in evading immune surveillance and promotes tumor growth and metastasis through anti−apoptotic and pro−proliferative effects (44, 45). However, its role in HGSOC has not been reported. Our study revealed that *BAG5* is upregulated in HGSOC, serving as a protective factor for patient survival. We propose that the ISR might be excessively activated in HGSOC, and *BAG5* upregulation could moderately modulate the ISR, thereby maintaining the “stress–adaptation” balance in tumor cells and delaying tumor progression. The role of *DNAJB1* in tumors is dual−pronged. On the one hand, it can inhibit tumor cell growth by suppressing abnormal protein accumulation, exerting a tumor−suppressive effect (46); on the other hand, it might be exploited by tumor cells to promote survival and drug resistance (47–50), with gastric cancer studies suggesting its association with platinum−based drug resistance (51). In this study, database analysis revealed that *DNAJB1* is highly expressed in HGSOC, and MR analysis supported its role as a risk gene, exhibiting relatively stable contributions to risk scores in prognostic models constructed from public data. However, validation using independent clinical samples via RT−qPCR, western blotting, and immunohistochemical staining indicated a trend of low expression, contradicting the database results. This discrepancy might have arisen from racial differences, variations in sample staging and processing methods, or the post−transcriptional and post−translational regulation of *DNAJB1* as a chaperone protein. Nevertheless, we included *DNAJB1* in the prognostic model: MR analysis provided genetic evidence for its causal relationship with HGSOC, and its identification as a risk gene is independent of absolute expression levels. In the GEO and TCGA datasets, the contribution of *DNAJB1* to risk scores was consistent across cohorts, demonstrating certain prognostic stratification value; moreover, the literature suggests that *DNAJB1* depletion in cancer cells can promote cancer cell growth (52). Therefore, despite the discrepancy in expression direction, the potential of *DNAJB1* as a prognostic biomarker and its possible role in platinum resistance in HGSOC warrant further investigation. Subsequent studies should clarify its mechanisms through functional experiments in multicenter cohorts.

*NUP35* plays a crucial role in fundamental cellular activities (53). Its aberrant expression in certain tumors can influence tumor cell proliferation, invasion, and metastasis (54–56), but its role in HGSOC has not been reported. This study found that *NUP35* is highly expressed in HGSOC, suggesting that it can delay tumor progression by modulating the ISR pathway. *CASP3* encodes a key protease in apoptosis and exhibits a dual role in cancer. It can induce tumor cell apoptosis (57), but its suppression or activation might also enable tumor cells to evade apoptosis (58). Previous studies indicated that *CASP3* activity is associated with platinum resistance in HGSOC (59), yet its independent prognostic value remains unclear. Our study demonstrated that *CASP3* is highly expressed in HGSOC, indicating its potential as an independent prognostic indicator and laying a foundation for exploring mechanisms of platinum resistance. In summary, this study suggests that *NUP35, CASP3*, and *BAG5* suppress HGSOC progression, whereas *DNAJB1* appears to promote HGSOC progression. Their dysregulated expression might be linked to an imbalance in ISR homeostasis, thereby influencing HGSOC progression and drug resistance. Future research should aim to further investigate these mechanisms.

To understand the potential mechanisms of the prognostic genes, this study conducted GSEA in the high- and low-risk groups and identified 15 differential KEGG pathways. These included the PPAR signaling pathway, primary immunodeficiency diseases, and systemic lupus erythematosus. The PPAR pathway regulates target gene expression and plays pivotal roles in energy metabolism, cell differentiation, inflammatory responses, and oxidative stress (60, 61). Additionally, it interacts with immune regulation within the tumor microenvironment (62). Different PPAR subtypes exhibit complex pro-tumorigenic or anti-tumorigenic functions in cancer, depending on the tumor type, subtype-specific expression patterns, and microenvironmental factors. Our study found significant differences in PPAR signaling pathway activity between the high- and low-risk groups. Therefore, we speculate that prognostic genes might influence cell fate and patient prognosis by regulating the ISR. An association exists between the ISR and PPAR signaling pathways, which might jointly participate in metabolic reprogramming and immune microenvironment regulation within tumors, thereby affecting patient prognosis.

Immune infiltration analysis revealed significant differences between the high− and low−risk groups for three immune cell types, including M2 macrophages and neutrophils. Previous studies confirmed that M2 macrophages mediate immunosuppression and tumor progression in HGSOC (11, 63), whereas neutrophil activity drives tumor development and drug resistance (64). Based on these findings, we speculate that these two cell types might influence the progression and prognosis of HGSOC, and targeting these cells or their secreted factors could represent a novel strategy to reverse the immunosuppressive microenvironment. Further analysis of immune checkpoint molecules indicated that the expression of *ICOS* and *IDO1* differed significantly between the high− and low−risk groups, and the TIDE score indicated a stronger response to immune checkpoint blockade in the high−risk group (P = 0.0083, **Figures 5A, B**). *ICOS*, as a T−cell co−stimulatory molecule, can enhance effector T−cell activity, whereas *IDO1* mediates immunosuppression by metabolizing tryptophan and promotes tumor immune escape (65). Therefore, targeting *IDO1* to inhibit suppressive signals and activating *ICOS* to enhance stimulatory signals might remodel the immune microenvironment of HGSOC and reverse immune evasion. Meanwhile, drug sensitivity analysis revealed that compounds such as ispinesib mesylate, KIN001−102, and navitoclax exhibited low IC_50_s in HGSOC, suggesting their potential therapeutic utility. In summary, these findings provide clues for immunotherapy and combination drug strategies in HGSOC, although further clinical studies are required for validation.

Finally, we conducted single-cell data analysis, identifying epithelial cells as the key cells. Epithelial cells drive the progression of malignant tumors through epithelial–mesenchymal transition, tumor microenvironment regulation, and genomic variations (66–68). HGSOC, the most aggressive subtype of ovarian cancer, is closely associated with the malignant transformation of fallopian tube secretory epithelial cells (68). TP53 mutations in epithelial cells (67, 68) and the malignant transformation of these cells are involved in HGSOC progression (66). Based on these findings, we speculate that the four prognostic genes might be associated with the ISR, which regulates cellular stress and genome stability, and they might influence the accumulation of *TP53* mutations or epithelial–mesenchymal transition in epithelial cells, thereby contributing to the risk of malignant transformation. These findings offer insights into prognosis assessment and therapeutic strategies for patients with HGSOC.

This study identified four ISR-related prognostic genes in HGSOC through public databases and, for the first time, constructed and validated a prognostic risk model for HGSOC based on ISR-RGs. This model might provide a reference for the prognostic evaluation of patients with HGSOC with aberrant ISR activation. Furthermore, we analyzed the expression profiles of these four prognostic genes in key cell types at the single−cell level, offering insights into their potential roles in HGSOC. However, this study had several limitations. First, as multivariate Cox regression analysis incorporating clinical variables was not performed, the independent prognostic value of the risk score remains uncertain, requiring further validation in multivariable models. Second, the confidence intervals of the HRs for *NUP35* and *BAG5* were relatively wide, indicating limited precision in effect estimates. Their robustness needs to be verified in larger external cohorts. Third, the underlying mechanisms of the ISR in HGSOC warrant additional experimental investigation. In subsequent work, we will further validate the expression patterns of these genes and the independent prognostic utility of the risk score using methods such as multivariate Cox regression and delve into their functional mechanisms within the ISR pathway.

## Conclusions

Through bioinformatic analysis of public databases, this study identified four prognostic genes (*NUP35*, *CASP3*, *BAG5*, and *DNAJB1*) and constructed a risk model. The model displayed prognostic predictive potential in HGSOC and suggested a degree of applicability in independent datasets. The risk score exhibited significant correlations with patient survival status, immune cell infiltration (e.g., M2 macrophages), and drug sensitivity. Single−cell analysis revealed the potential role of epithelial cells in HGSOC progression, with their differentiation trajectory associated with prognostic gene expression changes. This model provides a reference for individualized prognosis assessment and targeted therapy in HGSOC. Future experimental validation is required to elucidate the functional mechanisms of these genes within the ISR pathway.

## Data Availability

The original contributions presented in the study are included in the article/**Supplementary Material**. Further inquiries can be directed to the corresponding author.

## Supplementary material

Supplementary Figure S1 Forest plot of Mendelian randomization results. The first column shows genes with P <0.05 in IVW algorithm; the second column shows p values; the third column shows OR values, OR >1: Positive correlation (gene increases risk of the outcome), OR <1: Negative correlation (gene decreases risk of the outcome); and the fourth column shows OR values and 95% CI.

Supplementary Figure S2 Correlation analysis between exposure factors and outcome. S2 scatter plot of MR analysis. A positive slope of the line indicates that the exposure factor is a risk factor for the outcome variable, and vice versa.

Supplementary Figure S3 Diagnostic efficacy forest plot for exposure-outcome association. If the value is greater than 0, it indicates a positive causal relationship between the SNP locus and the outcome, and vice versa.

Supplementary Figure S4 Funnel plots for assessing the randomness of exposure factors. The funnel plot was constructed based on the effect values (β) and standard errors (SE) of individual instrumental variables, and the distribution pattern can be used to assess whether the analysis conforms to Mendel’s Second Law.

Supplementary Figure S5 Forest plot of the leave-one-out analysis. The meta-analytic effect of remaining SNPs after excluding each SNP individually. The red line indicates the overall meta-analytic effect.

Supplementary Figure S6 Expression of 4 prognostic genes at protein level in various cancers. The deeper the color, the higher the intensity of expression.

Supplementary Figure S7 Post-quality control violin plots showing distributions of nFeature_RNA, nCount_RNA, and percent.mt.

Supplementary Figure S8 Screening of highly variable genes (HVGs).

Supplementary Figure S9 Dimension reduction and clustering of single-cell data.

Supplementary Figure S10 Screen the key feature variables of the data.

Supplementary Figure S11 shows the scree plot of PCs, while S9 represents the p-value distribution of PCs, with values below the dashed line indicating non-significance.

Supplementary Figure S12 UMAP plot for clustering analysis of cells from all samples.

Supplementary Table S1 A comprehensive list of ISR-RGs.

Supplementary Table S2 Primer sequences used for qPCR.

Supplementary Table S3 Enriched GO terms for candidate genes in HGSOC.

Supplementary Table S4 Enriched KEGG pathways for candidate genes in HGSOC.

Supplementary Table S5 MR sensitivity analysis: Heterogeneity test for the 40 candidate genes.

Supplementary Table S6 MR sensitivity analysis: Horizontal pleiotropy test for the 40 candidate genes.

Supplementary Table S7 Results of the MR Steiger directionality test for the 40 candidate genes.

Supplementary Table S8 Results of the PH assumption test for the 39 candidate genes.

Supplementary Table S9 Significantly enriched pathways from the GSEA between the High-risk Group and Low-risk Group groups.

Supplementary Table S10 Correlation analysis between the expression of four prognostic genes (*NUP35*, *CASP3*, *BAG5*, *DNAJB1*) and the IC_50_ values of 138 drugs.

Supplementary Table S11 TFs-target gene regulatory relationships for the four prognostic biomarkers.
