# Supplementary figures and images for: Identification of integrated stress response-related prognostic genes in high-grade serous ovarian cancer using Mendelian randomization, single-cell RNA sequencing, and bulk RNA sequencing
Source: Front Oncol. 2026 May 5;16:1798083. doi: 10.3389/fonc.2026.1798083 (PMC13183660; doi:10.3389/fonc.2026.1798083)

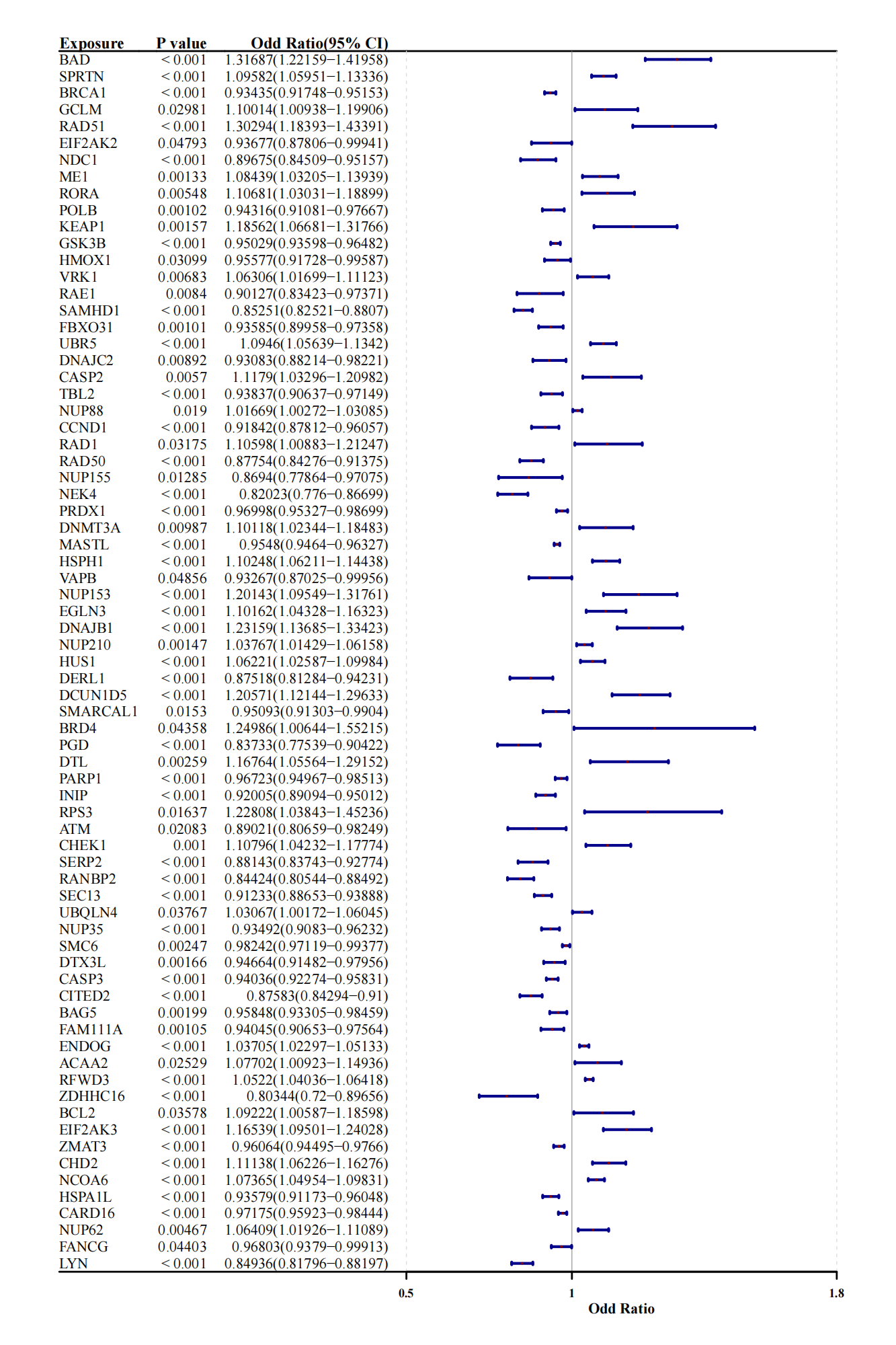

Supplement: Supplementary file 12 [file Image1.tif]

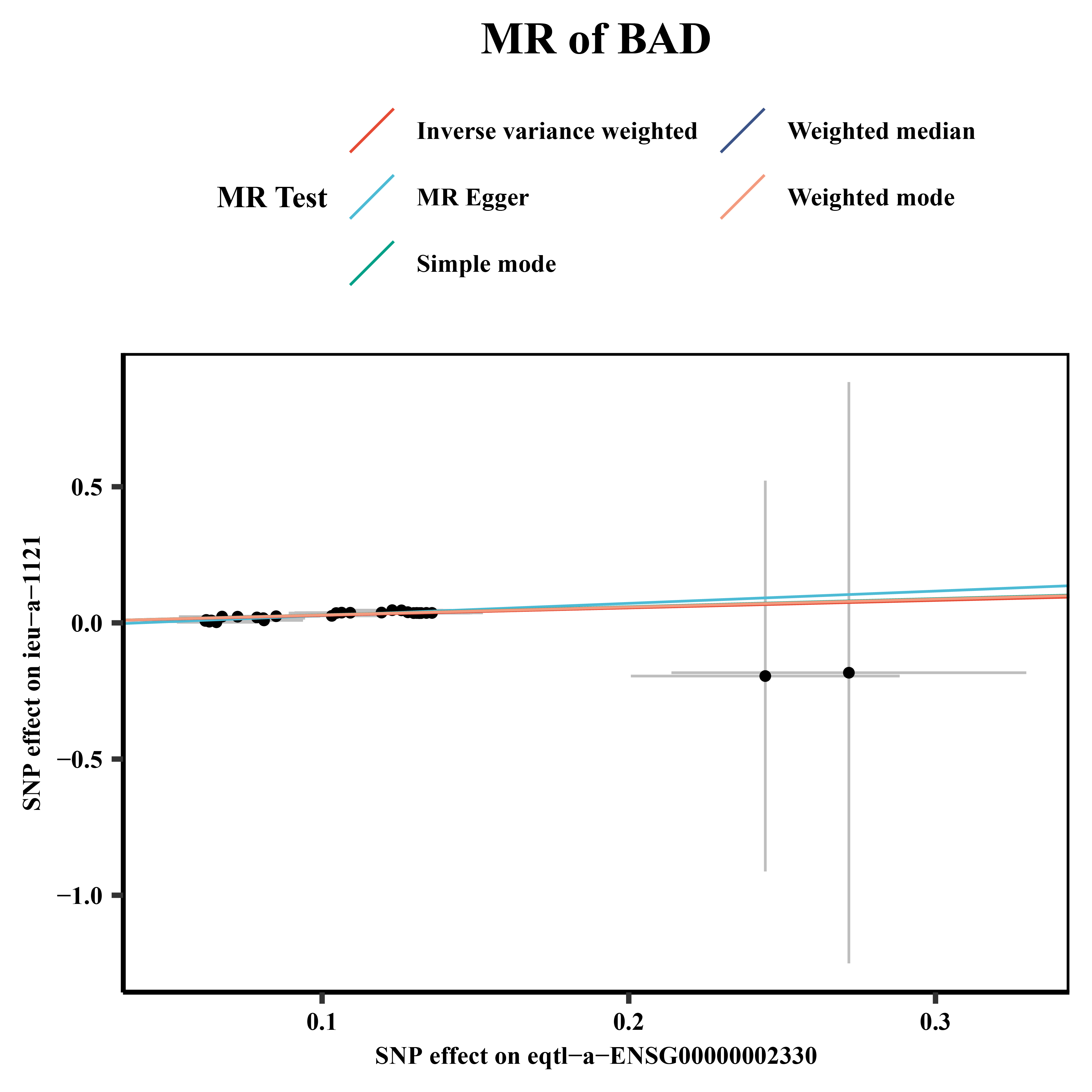

Supplement: Supplementary file 13 [file Image2.tif]

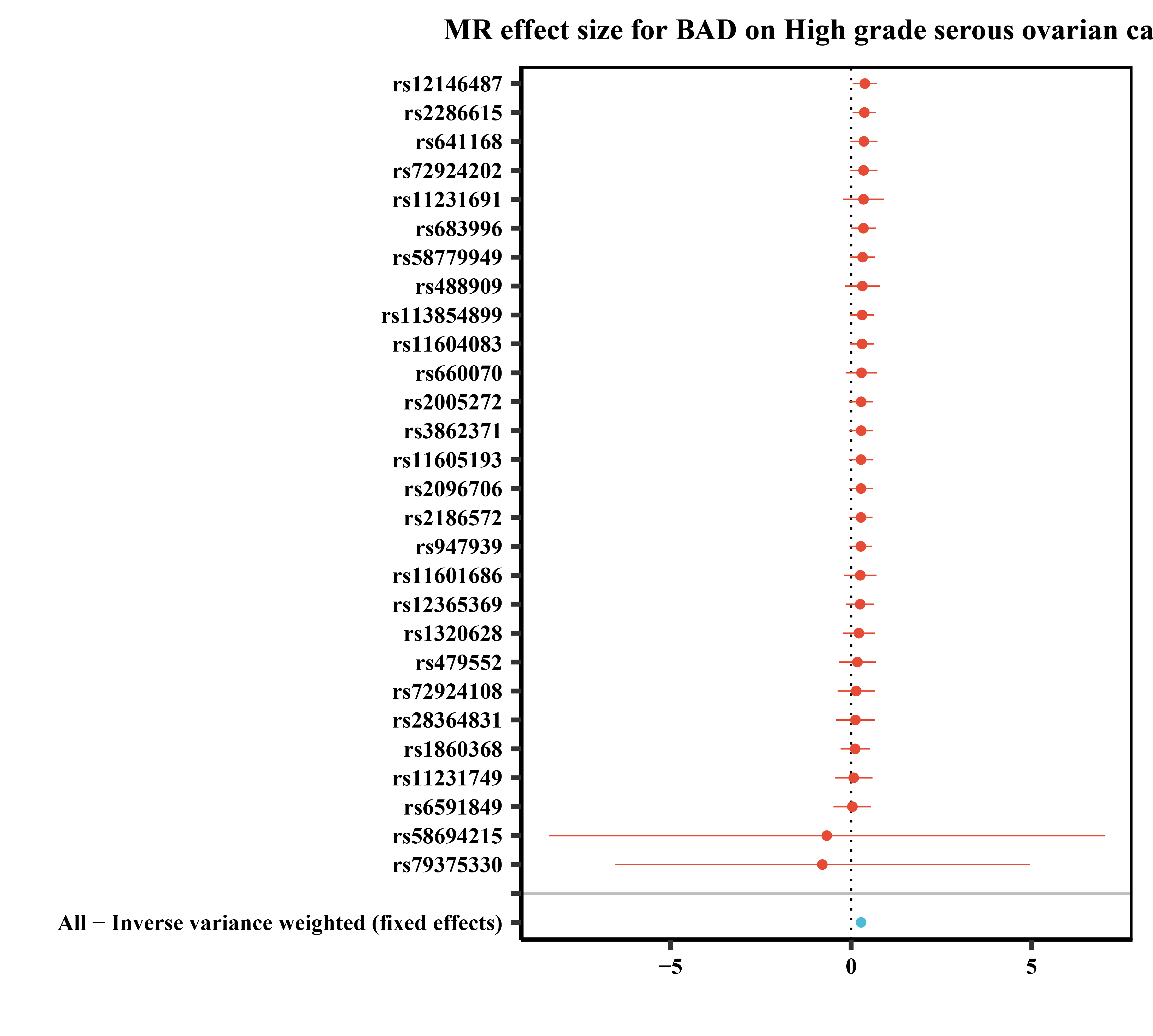

Supplement: Supplementary file 14 [file Image3.tif]

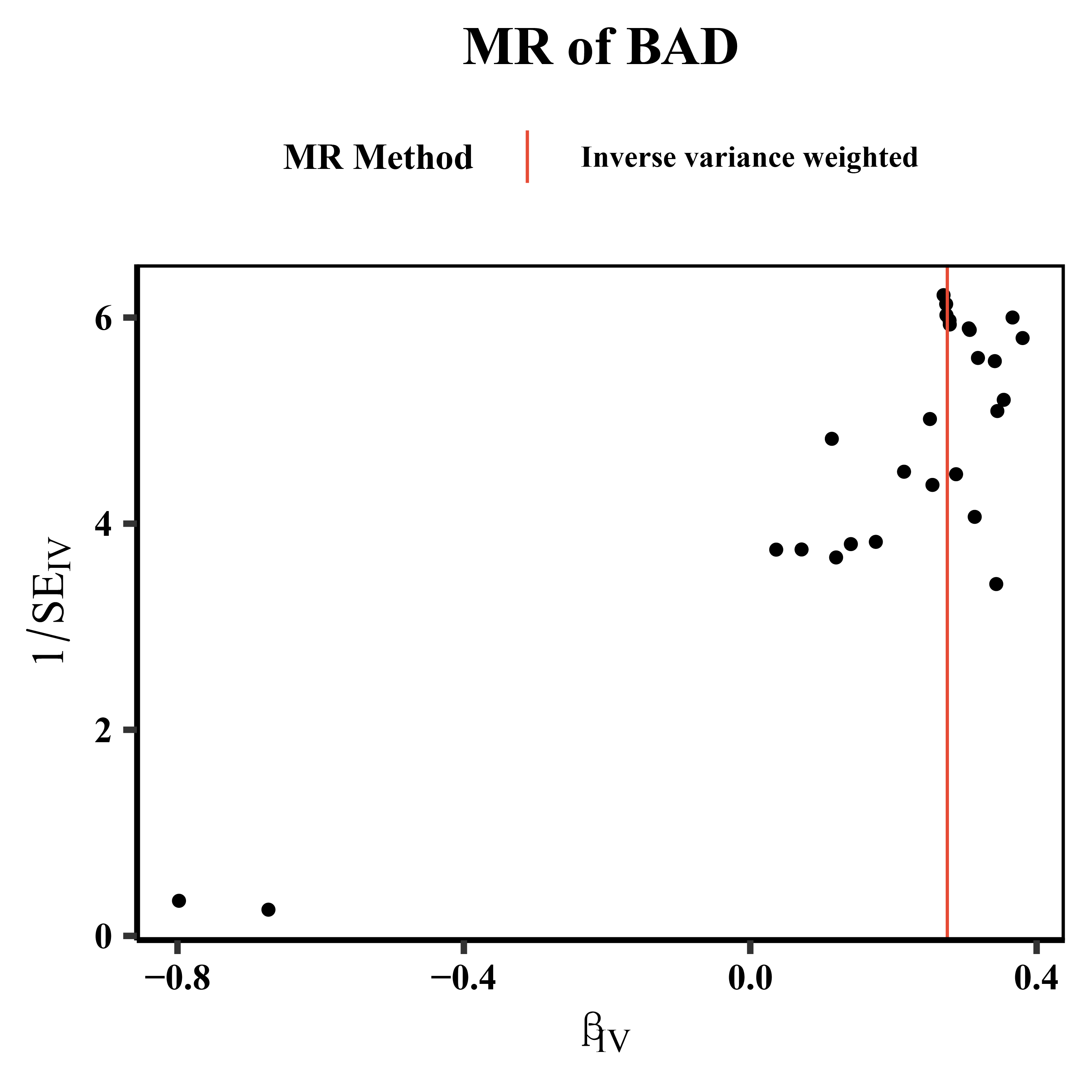

Supplement: Supplementary file 15 [file Image4.tif]

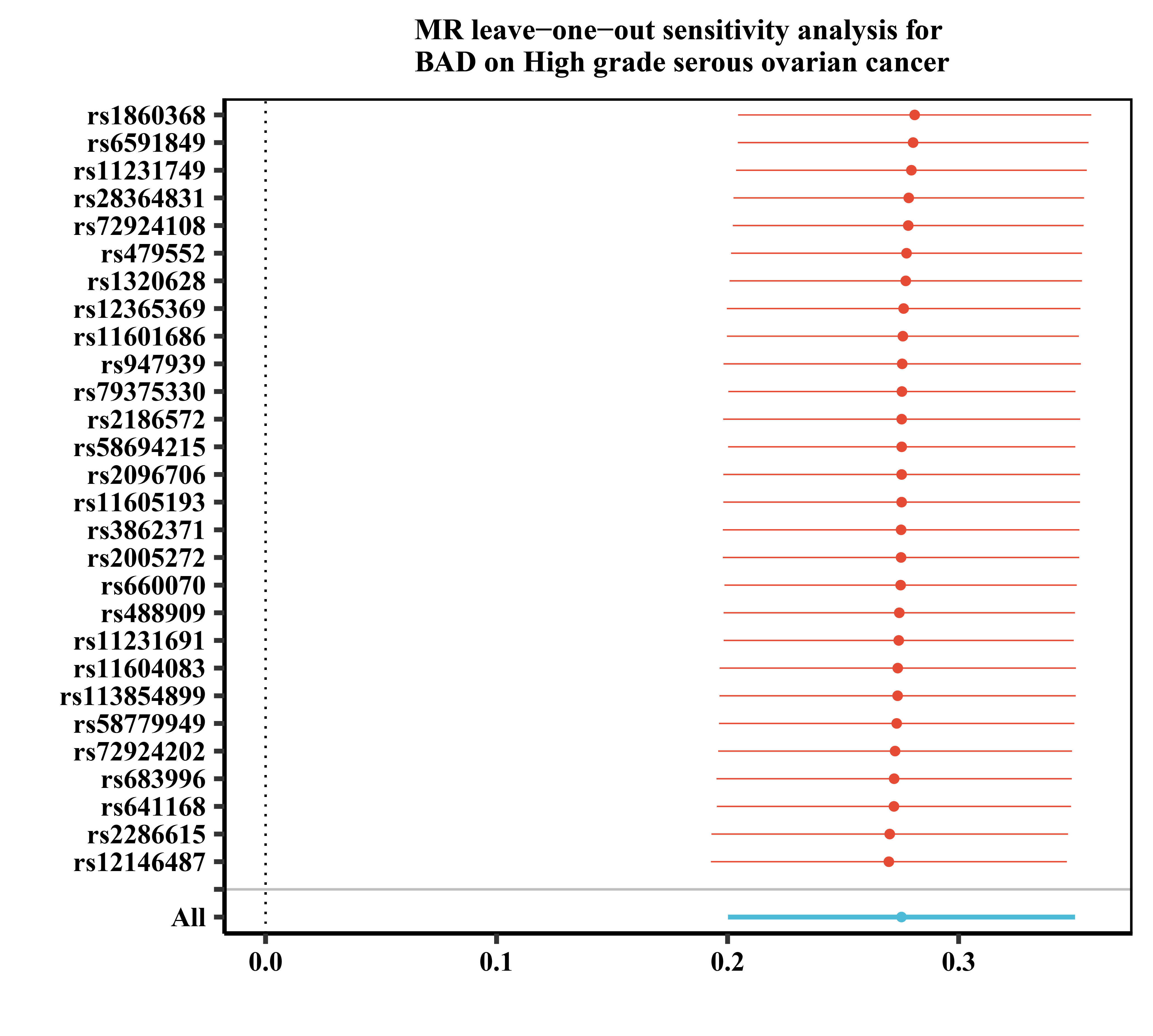

Supplement: Supplementary file 16 [file Image5.tif]

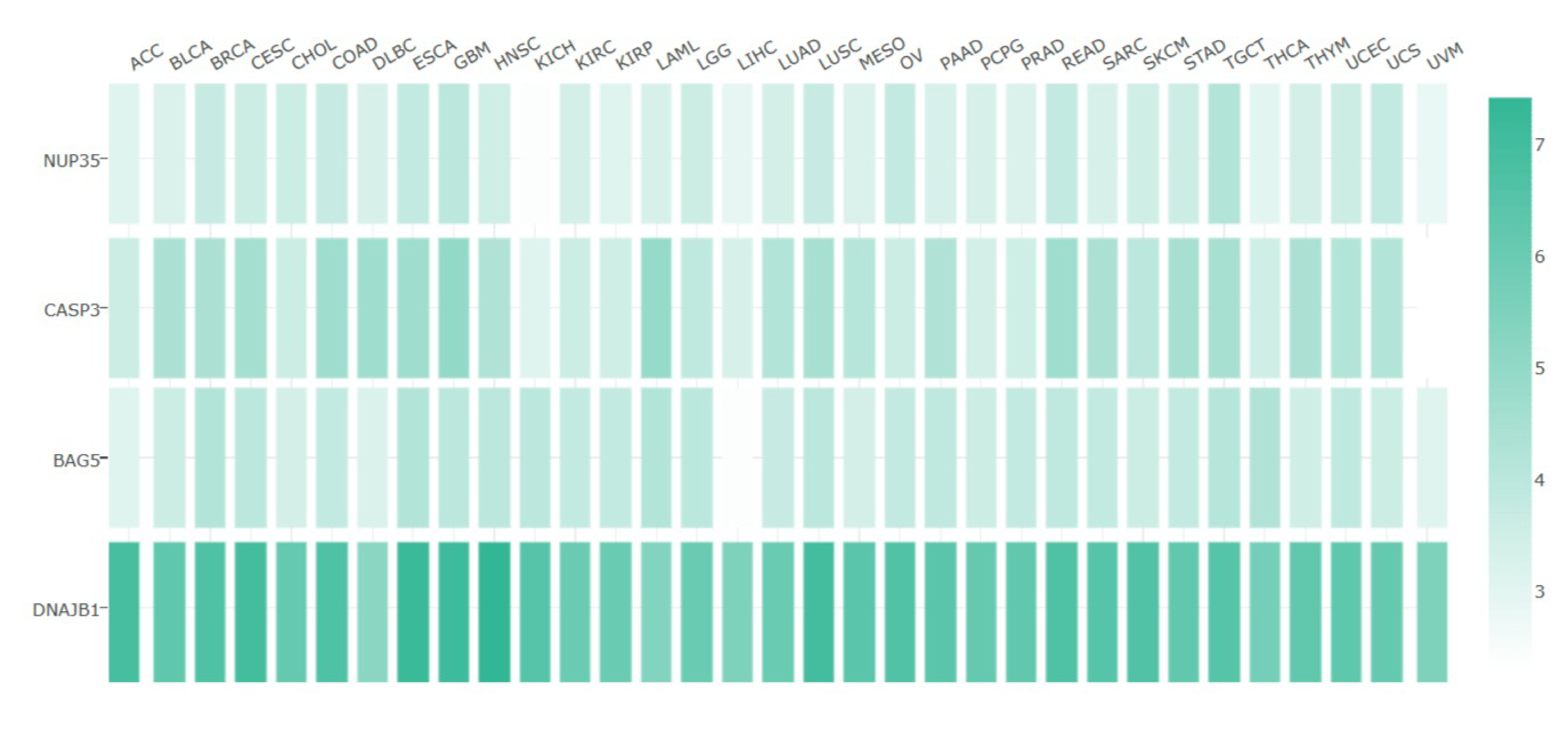

Supplement: Supplementary file 17 [file Image6.tif]

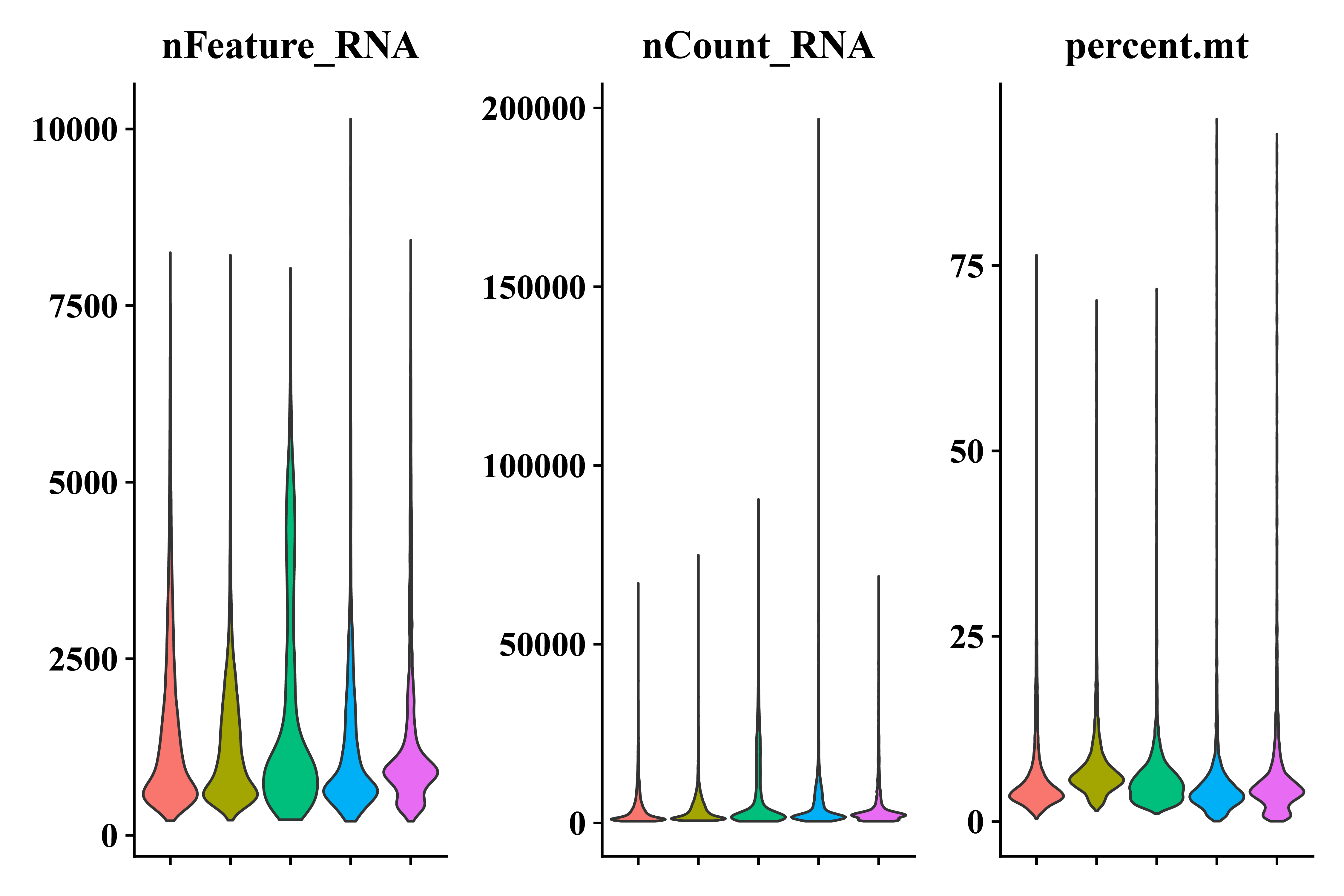

Supplement: Supplementary file 18 [file Image7.tif]

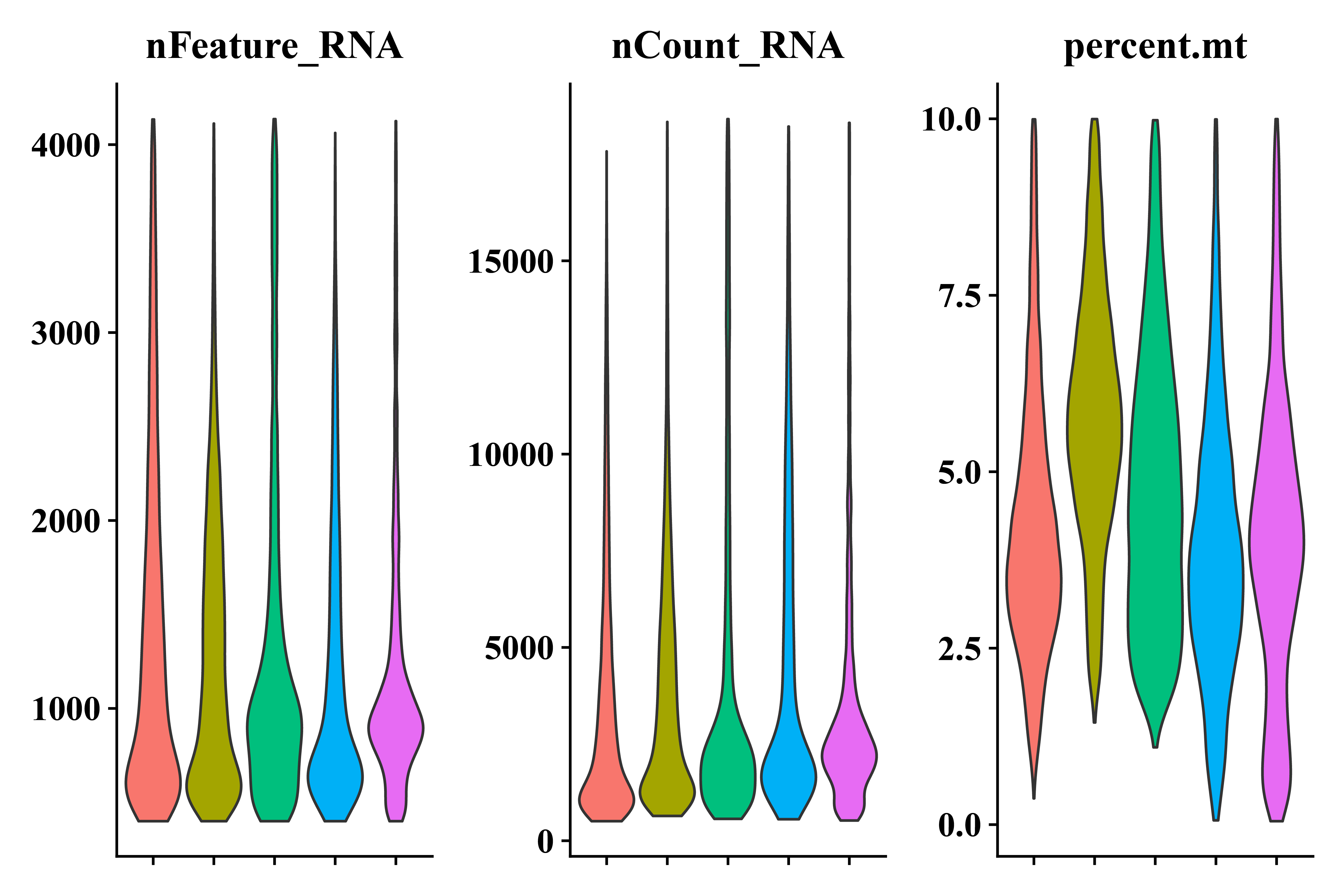

Supplement: Supplementary file 19 [file Image8.tif]

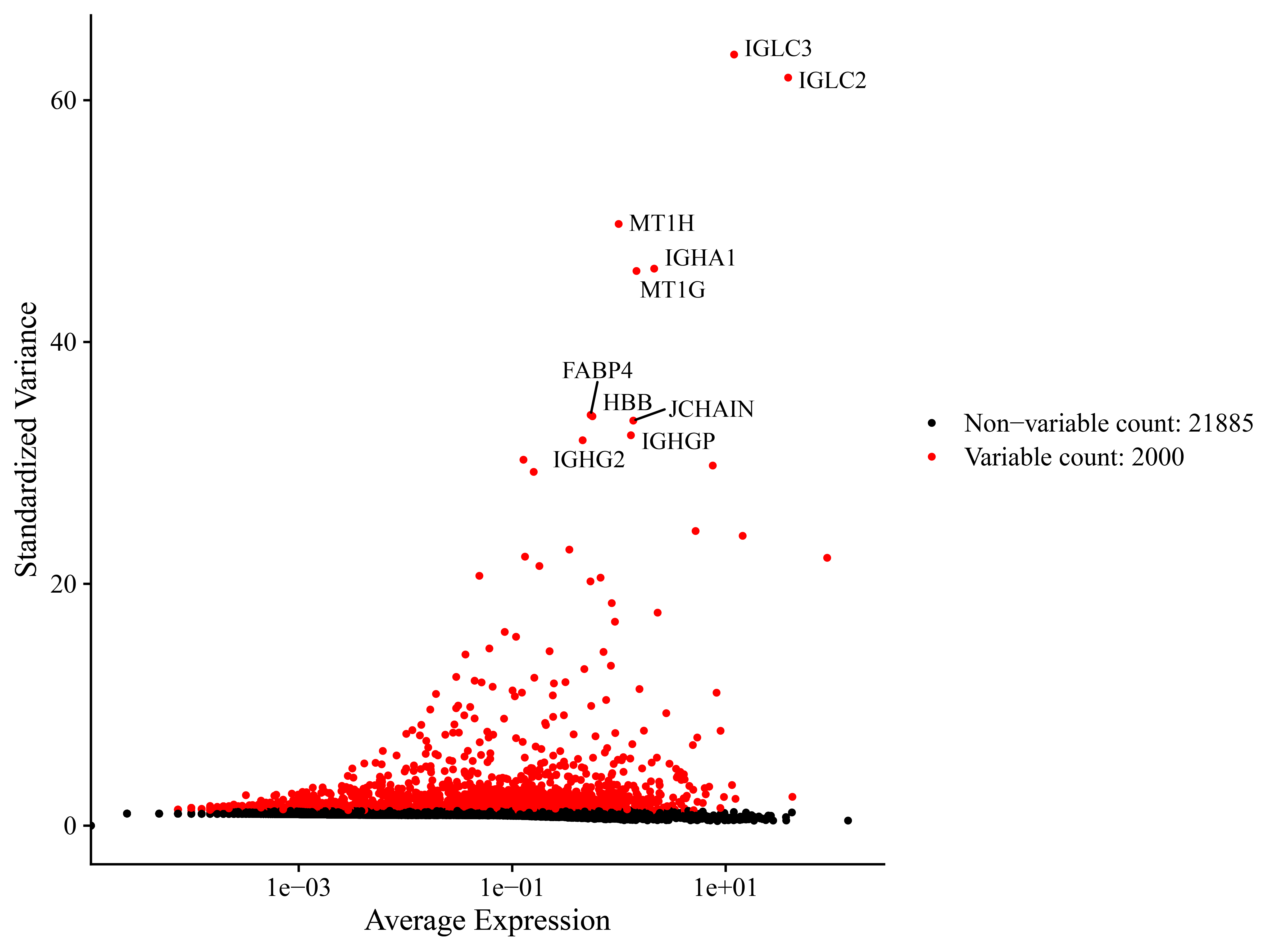

Supplement: Supplementary file 20 [file Image9.tif]

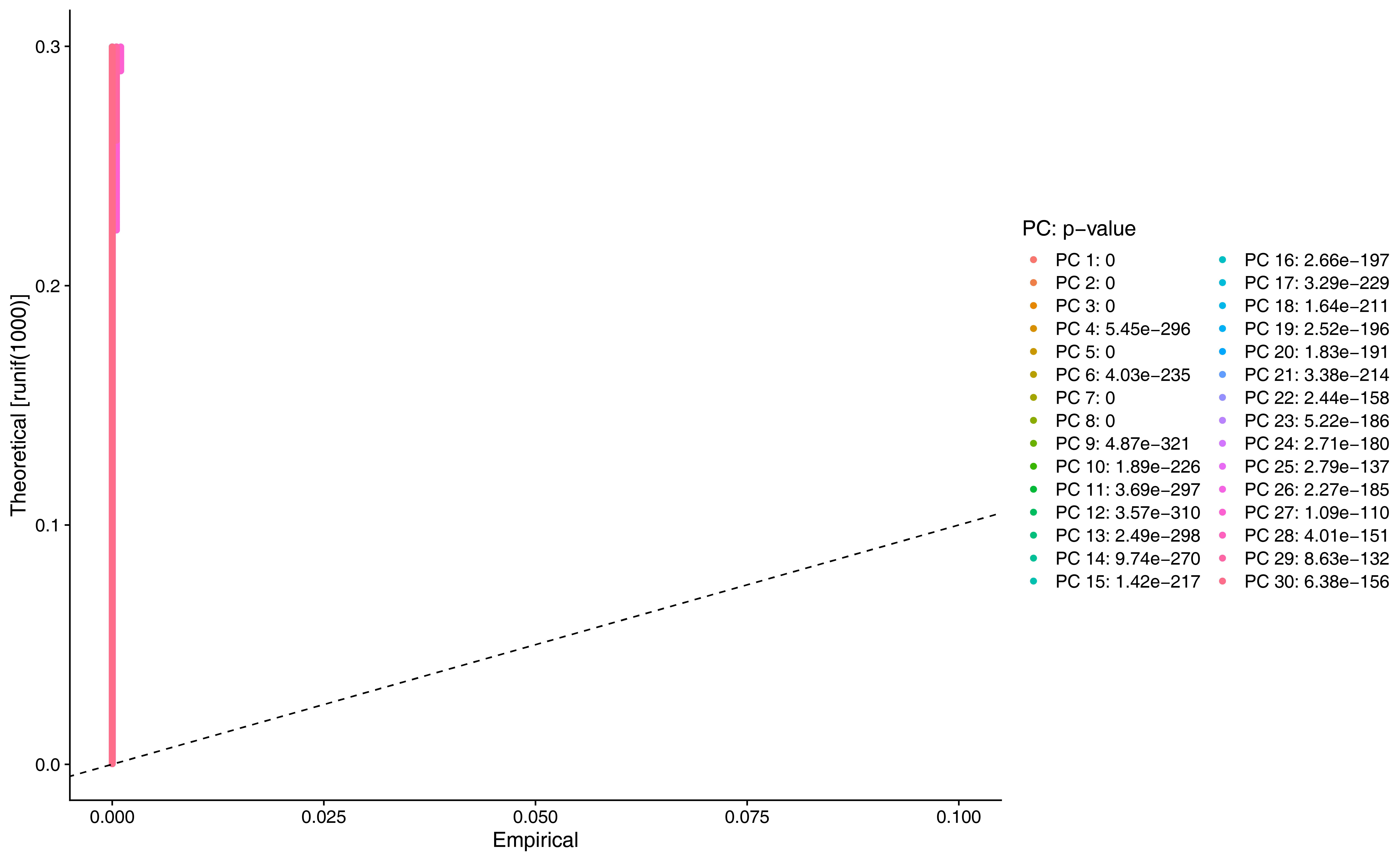

Supplement: Supplementary file 21 [file Image10.tif]

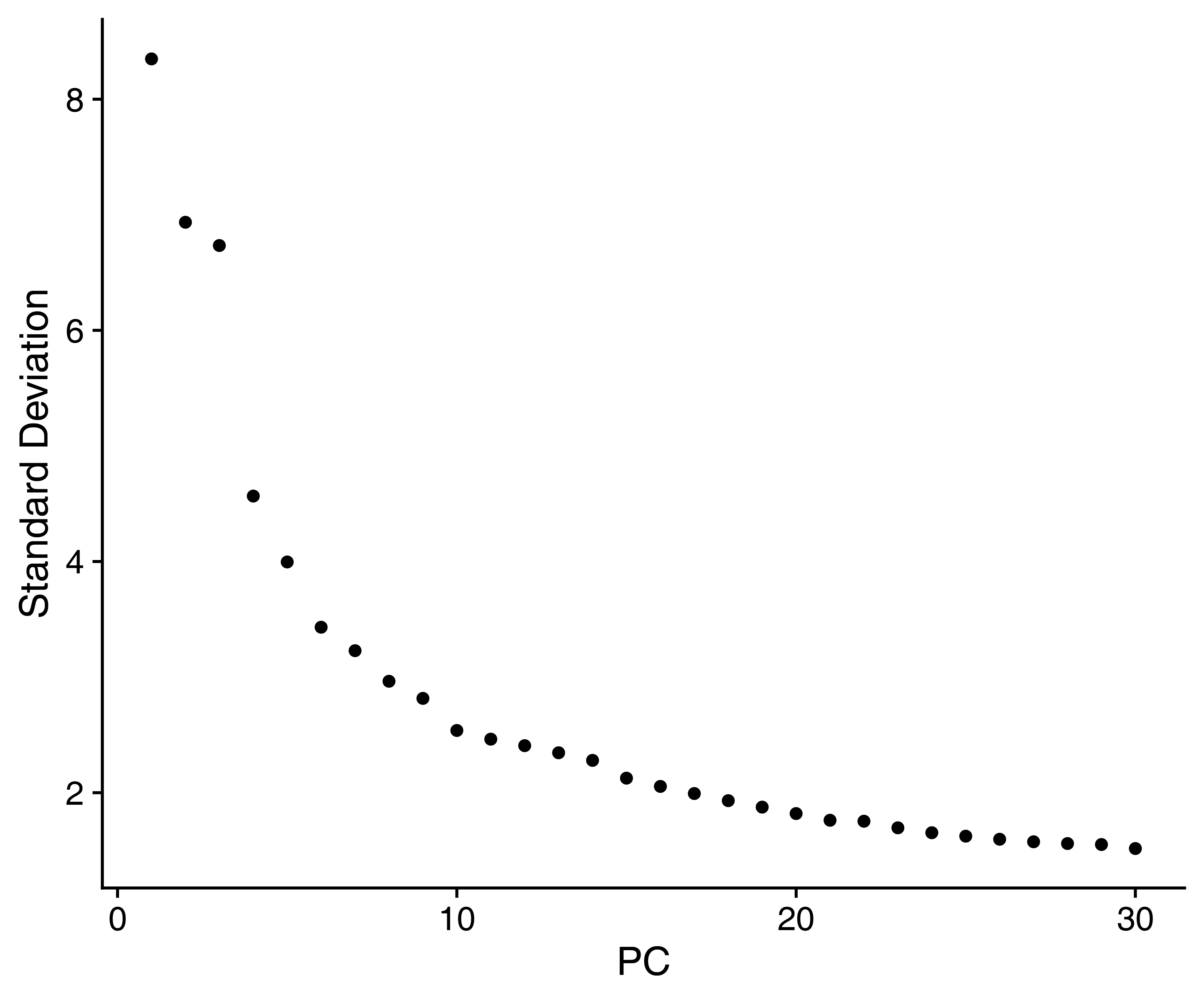

Supplement: Supplementary file 22 [file Image11.tif]

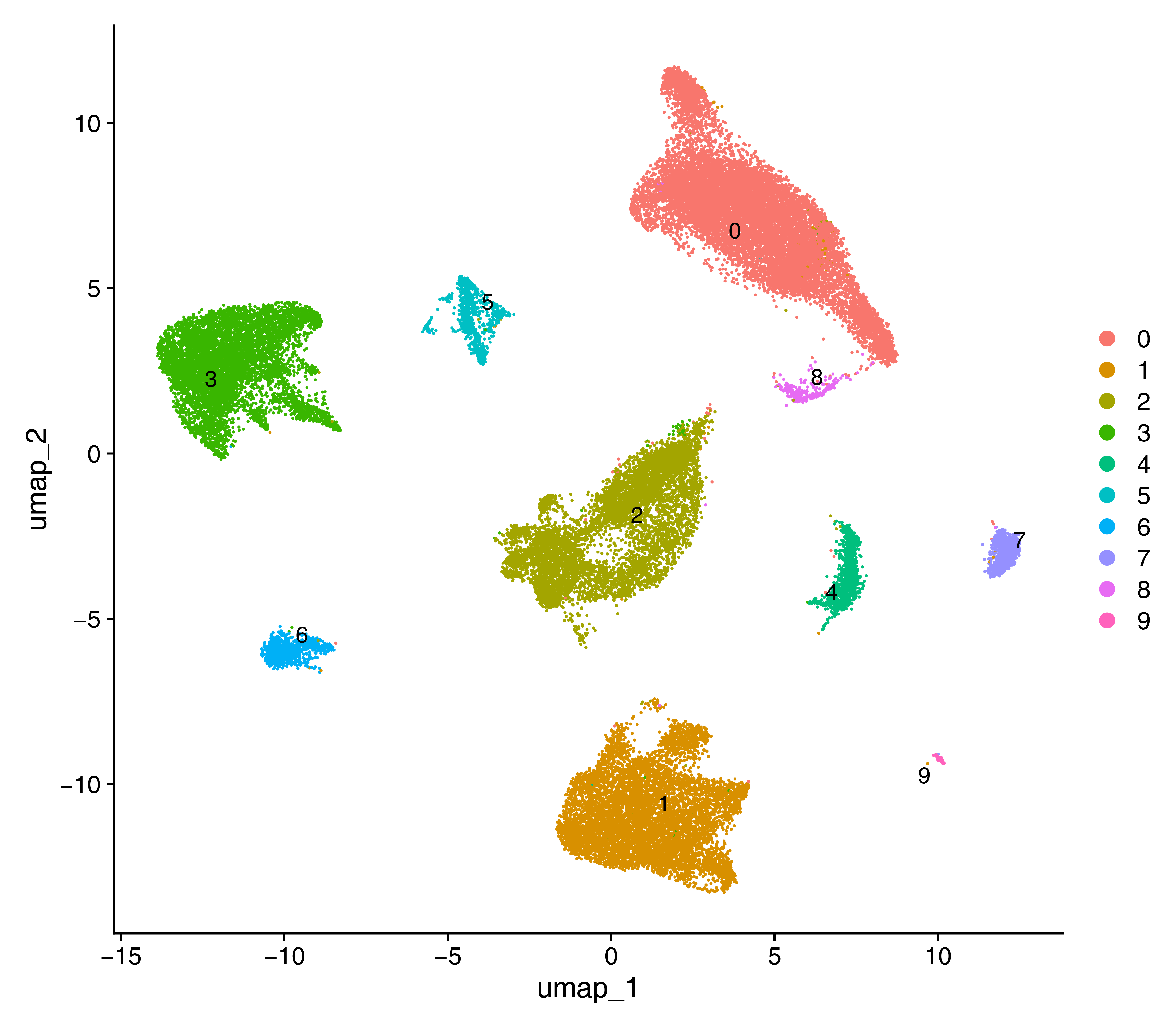

Supplement: Supplementary file 23 [file Image12.tif]

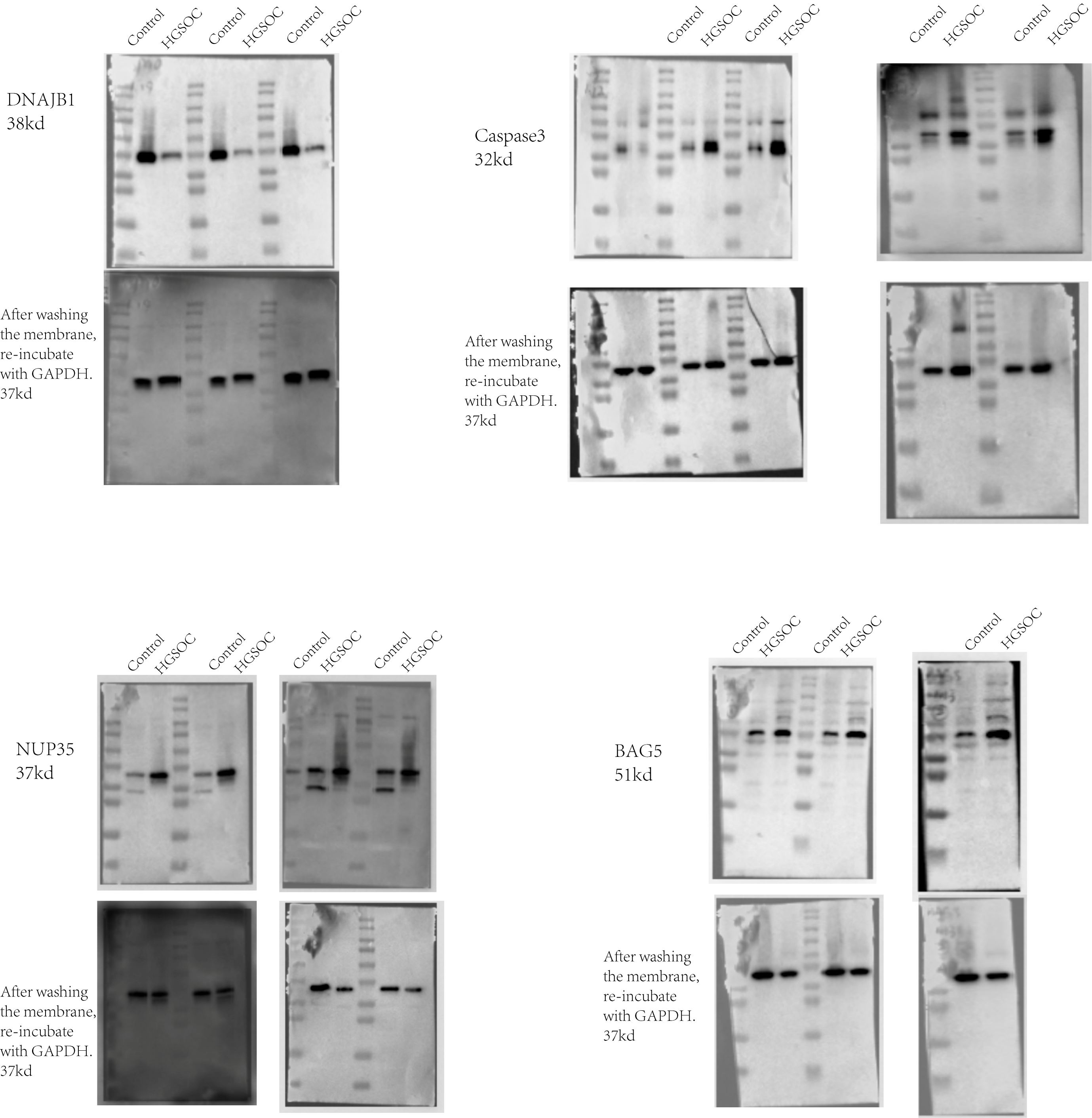

Supplement: Supplementary file 24 [file Image13.tiff]
